# Enteral feeding tube use among pediatric patients and nurses’ knowledge in China: a 52-hospital point-prevalence and cross-sectional survey study

**DOI:** 10.3389/fnut.2026.1854191

**Published:** 2026-08-18

**Authors:** Zhuowen Yu, Yuyan Huang, Yiwen Zhou, Tianchan Lyu, Yuxia Yang, Ying Wu, Yurong Zhang, Junhua Tian, Xiaofeng Xu, Siyi Wang, Beth Lyman, Jos M. Latour, Linqi Zhang, Ying Gu

**Affiliations:** 1 Department of Nursing, Children’s Hospital of Fudan University, Shanghai, China; 2 Department of Gastroenterology and Hepatology, Children’s Hospital of Fudan University, Shanghai, China; 3 Neonatal Intensive Care Unit, Children’s Hospital of Fudan University, Shanghai, China; 4 Pediatric Intensive Care Unit, Children’s Hospital of Fudan University, Shanghai, China; 5 General Surgery, Children’s Hospital of Fudan University, Shanghai, China; 6 Respiratory Department, Children’s Hospital of Fudan University, Shanghai, China; 7 Outpatient and Emergency Department, Children’s Hospital of Fudan University, Shanghai, China; 8 School of Nursing, Fudan University, Shanghai, China; 9 Nutrition Support Nurse Consultant, Kansas, MO, United States; 10 School of Nursing and Midwifery, Faculty of Health, University of Plymouth, Plymouth, United Kingdom; 11 Department of Nursing, Beijing Children’s Hospital, Capital Medical University, Beijing, China

**Keywords:** child, cross-sectional studies, enteral nutrition, feeding tube, nurses, pediatrics, prevalence

## Abstract

**Background:**

Enteral feeding tubes are widely used in pediatric patients, but tube-related complications remain a safety concern. Understanding current practice and nurses’ knowledge is essential for improving the safety of enteral feeding tube placement and guiding evidence-based pediatric care.

**Aim:**

To investigate the current status of enteral feeding tube placements in hospitalized children in China and to evaluate nurses’ knowledge and experience of enteral feeding tube management.

**Methods:**

A cross-sectional point-prevalence study was conducted between 4 and 20 December 2024 across 52 hospitals in China to identify hospitalized children with enteral feeding tubes. Data were collected by trained nurses. Concurrently, a survey was conducted among 11,787 pediatric nurses in the same hospitals. Descriptive statistics and binary logistic regression analysis were used.

**Results:**

A total of 2,875 children with enteral feeding tubes were included. The national prevalence of enteral feeding tube placement was 7.7% (2,875/37,114) of all hospitalized children. The highest rates were observed in general hospitals with pediatric departments (14.4%), followed by maternal and child health hospitals (11.6%) and children’s hospitals (6.4%). Pediatric intensive care units (PICUs) (39.2%) and neonatal units (35.5%) were the primary units where patients with enteral feeding tubes were located. Orogastric (54.9%) and nasogastric (41.1%) tubes constituted the majority of feeding tube types. Among 11,787 nurses, 91.0% reported gastric tube placement experience, yet only 19.2% knew the nasal-tip-earlobe-midpoint-umbilicus method for correct tube length measurement and 8.8% were aware of the recommended protocol when gastric contents cannot be aspirated from the tube. Only 7.0% had post-pyloric tube placement experience, and 51.6% were unaware of the recommended flushing frequency of every 4–6 h during continuous infusion. After adjustment, nurses’ correct responses showed item-specific associations with geographic region, hospital type, department type, and nurse-level characteristics. Nurses in East China and South China generally had higher odds of correct responses for most knowledge items, and nurses from general hospitals with pediatric departments and PICUs showed higher odds for selected items. Higher education level and managerial position were associated with correct responses to some items, whereas longer pediatric nursing experience was not consistently associated with higher knowledge accuracy. Bedside ultrasound and X-ray were the primary tools for post-pyloric tube placement guidance and post-placement verification, respectively.

**Conclusion:**

This nationwide study identified substantial and item-specific gaps in pediatric nurses’ evidence-based knowledge of enteral feeding tube management. Standardized institutional protocols and targeted evidence-based education tailored to clinical settings and specific knowledge deficits are needed to improve the safety and quality of pediatric enteral tube feeding care.

## 1 Introduction

Enteral nutrition, defined as the delivery of nutrients via tube feeding or specialized oral nutritional supplements, serves as the cornerstone nutritional support intervention for hospitalized children (1, 2). The safety and efficacy of tube feeding depends on proper tube placement and standardized care. Pediatric patients exhibit significantly higher rates of feeding tube-related complications such as malposition, dislodgement, and aspiration pneumonia, compared to adults due to their unique anatomical characteristics (3). These factors underscore the need for evidence-based nursing care to deliver safe and effective enteral feeding tube management in pediatric patients.

As nurses primarily place and maintain these enteral tubes, their knowledge base and practical skills directly influence nutritional safety and treatment outcomes. International guidelines are available with explicit recommendations for pediatric enteral nutrition practices (4, 5). These guidelines provide evidence of the tube insertion, placement confirmation, utilization, and maintenance protocols. Despite these guidelines, disparities in nursing knowledge and nursing training could contribute to the heterogeneity in delivering standardized evidence-based clinical practice.

The NOVEL (New Opportunities for Verification of Enteral tube Location) initiative, sponsored by the American Society for Parenteral and Enteral Nutrition (ASPEN), is an evidence-based program to identify practical solutions for nasogastric tube placement and tip placement verification (6). Inspired by this project, Lyman et al. conducted a nationwide 1-day prevalence study in 2015 to investigate the prevalence of temporary enteral access devices in hospitalized children and nursing practice (7). Of the 63 hospitals, 1995 (24%) children had a temporary enteral access device, of which 1,316 (66%) children had a nasogastric tube. These findings highlight the frequent practices of the enteral feeding tubes in children and underscore the importance of accurate tube placement and verification practices.

Building on the NOVEL program, the aim of this study is to examine the current status of enteral feeding tube placements in children admitted to Chinese hospitals and to evaluate the nurses’ knowledge and experience of enteral feeding tubes placements. This multi-center cross-sectional study has been designed to identify gaps between clinical practice and evidence-based recommendations. The evidence will support nurse training, ultimately providing safe and effective enteral tube feeding practice.

## 2 Materials and methods

### 2.1 Design and ethics

We used a multicenter, cross-sectional point-prevalence design to investigate the use of enteral feeding tubes in hospitalized children across mainland China and to assess pediatric nurses’ knowledge related to enteral tube placement and care. The Strengthening the Reporting of Observational Studies in Epidemiology (STROBE) guideline was used to report the study (8). The point-prevalence component captured eligible children with an enteral feeding tube hospitalized in 52 hospitals. Concurrently, a cross-sectional survey assessed nurses’ knowledge within the participating hospitals.

This study was approved by the Ethics Committee of the Children’s Hospital of Fudan University [No. (2024)311]. Written informed consent was obtained from all parents related to the patient-related data collection and all nurses participating in the survey before completing the survey.

### 2.2 Settings

**Figure 1:**
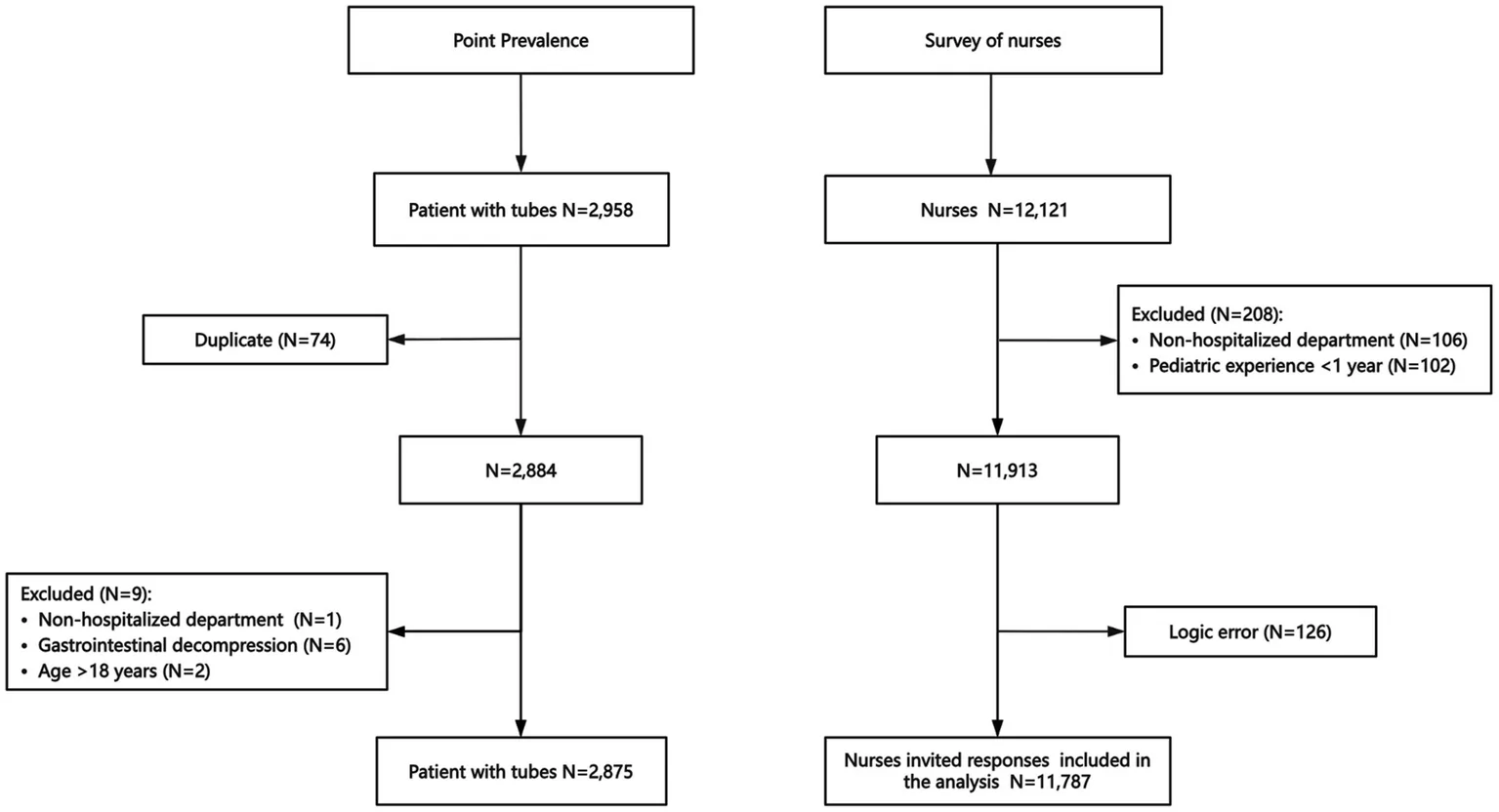
Study flowchart.

A total of 52 hospitals across 32 regions in mainland China were included (Figure 1). Of these hospitals, 32 were children’s hospitals, 14 were general hospitals with pediatric departments, and six were maternal and child health hospitals.

The point-prevalence study was conducted between 4 and 20 December 2024. During this period, each participating hospital collected point-prevalence data on one designated day. The nurse knowledge survey was distributed and completed during the same study period.

### 2.3 Participants

Inclusion criteria for children were: children from birth to 18 years of age who had an enteral feeding tube in place. Exclusion criteria were children with tubes not used for feeding, such as gastric decompression tubes (passive or active).

Inclusion criteria for nurses were: nurses who held a valid nursing license and had at least 1 year of pediatric clinical nursing experience. Exclusion criteria were nurses who were absent on the day of data collection or worked in pediatric wards that did not provide enteral feeding tube placement for children. Nurses in administrative or management roles and nursing trainees were also excluded.

### 2.4 Recruitment

As this was a nationwide multicenter descriptive point-prevalence study and cross-sectional survey, no formal sample size calculation was performed. Instead, the study size was determined by inviting all eligible hospitalized children with enteral feeding tubes during the study period and all eligible pediatric nurses from the participating hospitals. This approach was used to maximize the available study population and enhance national coverage and geographic diversity.

The multi-center recruitment initiative was officially launched in September 2024 during the Pediatric Nursing Academic Conference hosted by the Chinese Nursing Association. A preliminary screening channel was established via a digital platform. Interested institutions were instructed to scan a QR code to submit an electronic pre-survey form, which collected core parameters including institutional contact details, pediatric department resource configurations (available beds, registered nurses). Based on the information provided in the institutional pre-survey, hospitals were considered eligible if they provided pediatric inpatient services, had at least 200 pediatric beds and 150 registered pediatric nurses, performed more than 400 enteral feeding tube placements annually, and designated a local coordinator responsible for standardized data collection. Standardized invitation packages were then sent to all hospitals meeting these criteria. To broaden geographical coverage and reduce the underrepresentation of regions with relatively limited pediatric healthcare resources, additional hospitals were purposively invited based on their status as the principal tertiary pediatric care provider in their respective regions, even when they did not meet these eligibility criteria.

### 2.5 Instruments

#### 2.5.1 Point-prevalence data collection form

This instrument was designed to assess the current status of feeding tube use in hospitalized children, including: 1) Patient demographics: gender, age, department, height, and weight, hospital type. 2) Feeding tube characteristics: Types of tubes nasogastric tube (NGT), orogastric tube (OGT), post-pyloric tube (PP tube), gastrostomy tube, jejunostomy tube, percutaneous endoscopic gastrostomy with jejunal extension (PEG-J). 3) Department information: department category, total hospitalized children in the department (Electronic Supplementary Material S1).

#### 2.5.2 Pediatric nurses’ knowledge and experience survey

The survey was developed by the research team based on literature reviews (9–11) and consultations with hospital-based nutrition and feeding specialists to assess nurses’ knowledge of feeding tube placement, estimation of tube insertion depth, and confirmation of feeding tube position. Because G-tube, J-tube, and PEG-J placement falls outside the scope of nursing practice, this survey focused exclusively on NGT, OGT, and PP tube placement. The survey included two sections: Section 1 collected demographic data from nurses, including sex, age, professional title, position, and prior tube placement experience; Section 2 assessed knowledge and experience related to NGT/OGT/PP tube placement and tube-tip verification and included 15 questions (Electronic Supplementary Material S1). Of the 15 questions, nine were designed to assess knowledge of NGT/OGT/PP tube placement and management, and six were designed to explore nurses’ related clinical experience. Each question has 4 to 10 options. For the nine knowledge items, the evidence-based correct response was defined *a priori* by the research team on the basis of current guidelines and published literature used during questionnaire development. Each correct response was scored as 1 and each incorrect response as 0, yielding a total knowledge score ranging from 0 to 9; this total score was used only for reliability assessment and was not intended as the primary outcome measure of this study.

Content validity was assessed by seven experts who evaluated the content of the survey. The panel included two nurses with a PhD, two nurses with master’s degrees, and three nurses with bachelor’s degrees. Test–retest reliability was assessed among a subset of 60 nurses who completed the questionnaire twice at a two-week interval. The questionnaire demonstrated acceptable content validity and reliability for the purpose of this descriptive survey: S-CVI/UA, 0.867; S-CVI/Ave, 0.981; Cronbach’s alpha, 0.88. Among the 60 participants who completed the questionnaire twice at a 2-week interval, the ICC (A,1) for the total knowledge score was 0.830 (95% CI, 0.731–0.895). At the item level, observed agreement based on correct/incorrect scoring ranged from 81.7 to 100.0%, and Cohen’s kappa coefficients ranged from 0.641 to 1.000.

### 2.6 Data collection

Organizational Framework: Participating institutions were required to appoint one project leader and one coordinator. These researchers joined a dedicated WeChat group via QR code scanning to oversee site-specific training implementation and quality control.

Training: Training was organized for the hospital project leaders and coordinators from the 52 hospitals in October and November 2024. The first session provided a detailed interpretation of the study protocol and cross-sectional survey standards, while the second and third sessions covered operational guidelines for the research instruments, item-by-item survey clarification, and use of the electronic data capture system. After these sessions, local hospital leaders and coordinators trained ward coordinators on background data collection and quality assurance. Ward coordinators then informed all eligible clinical nurses about the study protocol and invited them to complete the survey.

Department-level coordinators collected inpatient feeding tube data, basic patient demographic information, and clinical data extracted from hospital and nursing information systems. The nurse survey was completed electronically by eligible nurses.

### 2.7 Quality control

Quality control was ensured through three integrated strategies: 1) an iterative survey development process combining theoretical frameworks, evidence, and clinical applicability; 2) implementation of standardized training programs to ensure consistency in data collection procedures across hospitals. Each participating hospital designated a trained project leader and coordinator to supervise local data collection, ensure adherence to the standardized study protocol, and verify data quality before submission; 3) a multi-stage validation protocol encompassing temporal screening of the online surveys, internal consistency verification, missing data management, logical coherence assessment, duplicate submission control, and centralized data verification. Specifically, online survey responses with a completion time <30 s or >30 min were excluded; contradictory responses within measurement dimensions were removed; questionnaires with critical incomplete items were flagged and excluded if unrecoverable; implausible responses were returned to the participating hospitals for verification against original source records; and duplicate submissions were controlled by retaining only the most recent valid entry. Verified discrepancies were corrected, whereas records that could not be verified were excluded from the final analysis.

### 2.8 Data analysis

Data were analyzed using IBM-SPSS version 26.0. Descriptive statistics summarized patient demographics, nurse demographics and feeding tube-related characteristics. Continuous variables are presented as ranges (min-max), and categorical variables are expressed as frequencies and percentages [*N*, (%)].

For the nurse knowledge survey, knowledge accuracy was first descriptively summarized across different levels of nurse and institutional characteristics, including geographic region, hospital type, department type, sex, age group, professional title, position, education level, and years of pediatric nursing experience. Each of the nine knowledge items was then analyzed separately as a binary outcome, with correct responses coded as 1 and incorrect responses as 0. Univariable logistic regression analyses were performed to examine crude associations between candidate factors and correct responses, followed by multivariable binary logistic regression models to identify factors independently associated with correct responses for each item. Crude odds ratios, adjusted odds ratios, 95% confidence intervals, and *p* values were reported, with a two-sided p value <0.05 considered statistically significant.

## 3 Results

### 3.1 Participants

The screening workflow for data collection is detailed in Figure 1. Of the 52 participating hospitals, 998 wards were included in the point-prevalence study. The total observed hospitalized children were 37,114. Of these, 2,875 (7.7%) were included in the point-prevalence analysis. The included children showed wide-ranging demographic variables: age 0–6,713 days, height 27.55–188 cm, and weight 0.47–90 kg with the median values being 1.36 years, 46 cm, and 2.6 kg, respectively. Totally, 12,121 nurses were invited to complete the knowledge survey. Of these, 11,787 (97%) completed the survey (Figure 1).

### 3.2 Children with enteral feeding tubes

Of the 37,114 hospitalized children on the data collection day, 2,875 children with enteral feeding tubes were recorded, corresponding to a prevalence of 7.7% (Table 1). Among these children, 2,888 devices were documented. The use of enteral feeding tubes was highest in South China (13.3%), Central China (8.9%), and Southwest China (8.3%). Institutional comparisons showed higher rates of children with an enteral feeding tube in general hospitals with pediatric departments and maternal and child health hospitals than in children’s hospitals (Table 1). The Pediatric Intensive Care Unit and neonatal unit were the primary clinical settings for feeding tube placement, with prevalence rates of 39.2 and 35.5%, respectively. Of the included children, most were male (1,674/2,875, 58.2%) and most were admitted to a neonatal unit (1,858/2,875, 64.6%). The most common primary diagnostic categories were preterm and term neonatal diseases (977/2,875, 34.0%), respiratory diseases (916/2,875, 31.9%) (Table 2). Most documented feeding tubes were OGTs (1,578/2,888, 54.9%) or NGTs (1,182/2,888, 41.1%) (Table 2).

**Table 1:** Prevalence of children with enteral feeding tubes.

Variables		Hospitalized children (*N*)	Children with tube* *N* (%)
All hospitals		37,114	2,875 (7.7)
Hospital type	CH	30,212	1941 (6.4)
	MCHH	2,165	252 (11.6)
	GHP	4,737	682 (14.4)
Region	Northeast	3,391	212 (6.3)
	North	7,208	492 (6.8)
	East	9,212	711 (7.7)
	South	2,819	375 (13.3)
	Central	5,389	478 (8.9)
	Northwest	5,049	270 (5.3)
	Southwest	4,046	337 (8.3)
Department	Neonatal unit	5,238	1858 (35.5)
	PICU	1,616	634 (39.2)
	Others	30,260	383 (1.3)

* Children with enteral feeding tube.

CH, Children’s hospitals; MCHH, Maternal and child health hospitals; GHP, General hospitals with pediatric departments.

**Table 2:** Patient demographic and clinical characteristics.

Variables (*N*, 2,875)		N	%
Gender	Male	1,674	58.2
	Female	1,201	41.8
Department	Neonatal unit	1,858	64.6
	PICU	634	22.1
	Others	383	13.3
Feeding tube type (*N*, 2,888)	NGT	1,182	41.1
	OGT	1,578	54.9
	PP tube	90	3.2
	Gastrostomy tube	21	0.7
	Jejunostomy tube	14	0.5
	PEG-J	3	0.1
Hospital types	GHP	682	23.7
	MCHH	252	8.8
	CH	1941	67.5
Primary diagnosis	Preterm and term neonatal diseases	977	34.0
	Respiratory diseases	916	31.9
	Circulatory system diseases	219	7.6
	Digestive system diseases	320	11.1
	Neurological diseases	290	10.1
	Hematological diseases	36	1.3
	Oncological diseases	27	0.9
	Other diseases	90	3.1

Neonatal unit, general neonatal unit and NICU; NGT, nasogastric tube; OGT, orogastric tube; PP tube, post-pyloric tube; PEG-J, percutaneous endoscopic gastrostomy with jejunal extension; CH, Children’s hospitals; MCHH, Maternal and child health hospitals; GHP. General hospitals with pediatric departments.

### 3.3 Knowledge and experiences of nurses

A total of 11,787 pediatric nurses aged 33.12 ± 6.38 (years), with 10.6 ± 6.72 years of clinical experience participated in the study (Table 3). While 91% (*N*, 10,722) reported gastric tube placement experience, only 8.8% (*N*, 1,036) demonstrated awareness of guideline-recommended protocols for managing unsuccessful gastric content aspiration.

**Table 3:** Nurse demographic and professional characteristics.

Variables (*N*, 11,787)		*N*	%
Gender	Male	386	3.3
	Female	11,401	96.7
Age	18–25	1,394	11.8
	26–30	2,964	25.1
	31–35	3,556	30.2
	36–40	2,537	21.5
	>40	1,336	11.3
Region	Northeast	951	8.1
	North	2,119	18
	East	3,092	26.2
	South	1,251	10.6
	Central	2065	17.5
	Northwest	1,155	9.8
	Southwest	1,154	9.8
Hospital type	CH	9,732	82.6
	MCHH	661	5.6
	GHP	1,394	11.8
Department	MW	5,156	43.7
	MSW	234	2.0
	SW	2,699	22.9
	Neonatal unit	2,191	18.6
	PICU	1,507	12.8
Professional title	None	1825	15.5
	Senior Nurse	4,890	41.5
	Supervisor Nurse	4,649	39.4
	Associate Chief Nurse+	423	3.6
Position	(Deputy) Head Nurse	622	5.3
	Clinical Nurse	11,165	94.7
Education	Associate Degree	1,162	9.9
	Bachelor’s Degree	10,418	88.4
	Post-graduate Degree	207	1.8
Pediatric working experience	1 ~ 5	2,866	24.3
	6 ~ 10	3,504	29.7
	11 ~ 20	4,557	38.7
	>20	860	7.3

CH, Children’s hospitals; MCHH, Maternal and child health hospitals; GHP, General hospitals with pediatric departments; MW, Medical ward; MSW, Medical and surgical ward; SW, Surgical ward.

The Hairline→Xiphoid (HX) method was the primary method used by nurses for external measurement before NGT placement (*N* = 6,067, 51.47%). The most commonly used methods for determining tube-tip position were aspiration (*N* = 11,237, 95.3%), auscultation (*N* = 10,634, 90.2%), and bubble observation (*N* = 9,623, 81.6%). Most nurses performed tube-tip verification before feeding (*N* = 11,300, 95.9%) and before shifts (*N* = 6,508, 55.2%) (Table 4).

**Table 4:** NGT/OGT knowledge and experience of nurses.

Variables (*N*, 11,787)		*N*	%
NGT/OGT tube placement experience	Yes	10,722	91.0
	No	1,065	9.0
Pre-insertion tube length measurement (NGT)	HX	6,067	51.47
	NEX	3,327	28.23
	NEMU	2,257	19.15
	Others	31	0.26
	Not clear	105	0.89
Tube tip verification methods	Aspiration of contents	11,237	95.3
	Visual inspection of aspiration color	3,107	26.4
	pH testing of aspiration	4,126	35.0
	Auscultation	10,634	90.2
	Bubble observation	9,623	81.6
	X-ray	4,702	39.9
	Ultrasound	2,218	18.8
	Not clear	26	0.2
	Endoscope	16	0.1
	Others	7	0.1
Aspiration pH	≤5.5	8,179	69.4
	5.6—10	1,325	11.2
	>10	57	0.5
	Not clear	2,226	18.9
Non-aspiration protocol	Positional adjustment with reaspiration	10,200	86.5
	Air insufflation	5,331	45.2
	X-ray	4,359	37.0
	Withdrawal and replacement	5,525	46.9
	Not clear	107	0.9
	Others	91	0.8
Tube tip verification timing (except dislodgement suspicion)	Pre-feeding	11,300	95.9
	Per shift	6,508	55.2
	Everyday	2,167	18.4
	Every week	1,064	9.0
	Placement confirmed, no further checks	80	0.7
	Others	164	1.4
	Not clear	94	0.8

NGT, Nasogastric tube; OGT, Orogastric tube; HX, Hairline → Xiphoid; NEX, Nasal tip → Earlobe → Xiphoid; NEMU, Nasal tip → Earlobe → Midpoint from xiphoid to umbilicus.

Only 7.0% (*N* = 826) of nurses had experience placing post-pyloric (PP) tubes. A total of 54.7% (*N* = 6,448) of nurses were not aware of methods for pre-insertion tube length measurement for nasojejunal tubes (NJT). Additionally, 48.2% selected X-ray as the method for tube-tip verification. Regarding previously used auxiliary devices for PP tube placement, 55.9% (*N* = 462) reported using bedside ultrasound. For verification of PP tube placement, X-ray was the most commonly used method, reported by 67.2% (*N* = 555) of nurses (Table 5).

**Table 5:** PP tube knowledge and experience of nurses.

Variables (*N*, 11,787)		*N*	%
Tube placement experience	Yes	826	7
	No	10,961	93
Pre-insertion tube length measurement (NJT)	NEX → Left/right iliac crest	551	4.7
	NEMU→Left/right iliac crest	1,173	10.0
	NEX → Left/right costal margin	417	3.5
	NEMU → Left/right costal margin-midaxillary line intersection +5–10 cm	3,138	26.6
	Not clear	6,448	54.7
	Others	60	0.5
Tube tip verification methods	Aspiration of contents	3,746	31.8
	pH testing of aspiration	4,184	35.5
	Visual inspection of aspiration color	2,776	23.6
	Auscultation	2,401	20.4
	Aspirate volume < Infuse volume	1899	16.1
	X-ray	5,685	48.2
	Ultrasound	3,017	25.6
	Endoscope	6	0.1
	Others	17	0.2
	Not clear	4,543	38.5
Aspiration pH	≤5.5	933	7.9
	5.6–7	2,468	20.9
	>7	3,024	25.7
	Not clear	5,362	45.5
Flushing frequency (continuous infusion)	Every 4-6 h	5,702	48.4
	Every 12 h	743	6.3
	Everyday	775	6.6
	Every week	48	0.4
	Not clear	4,435	37.6
	Others	84	0.7
Flushing liquid	Tap water	138	1.2
	Warm boiled water	5,524	46.9
	0.9% NaCl	2,653	22.5
	Sterile water for injection	471	4.0
	Not clear	2,997	25.4
	Others	4	0.0
Tube tip verification timing (except dislodgement suspicion)	Pre-feeding	7,877	66.8
	Per shift	5,118	43.4
	Everyday	1781	15.1
	Every Week	927	7.9
	Placement confirmed, no further checks	238	2.0
	Others	98	0.8
	Not clear	3,016	25.6
Previously used auxiliary devices for PP tube placement (*N*, 826)	Bedside ultrasound	462	55.9
	Endoscopy	286	34.6
	Electromagnetic guidance	90	10.9
	Blind insertions	433	52.4
	X-ray	8	1
Previously used verification devices for PP tube placement (*N*, 826)	X-ray	555	67.2
	Bedside ultrasound	490	59.3
	Endoscopy	265	32.1
	Electromagnetic guidance	76	9.2
	Auscultation	26	3.1

NJT, Nasojejunal tube; NEX, Nasal tip→Earlobe→Xiphoid; NEMU, Nasal tip→Earlobe→Midpoint from xiphoid to umbilicus; PP tube, Post-pyloric tube.

Nurses’ knowledge accuracy rates regarding NGT, OGT, and PP tube placement and care according to demographic and professional characteristics (region, hospital type, department, sex, age, professional title, position, education level, and years of pediatric nursing experience) are presented in Table 6. To further quantify these associations and adjust for potential confounders, univariable and multivariable logistic regression analyses were performed for each of the nine knowledge items. After adjustment, several regional, institutional, ward-level, and nurse-level characteristics remained associated with correct responses (Electronic Supplementary Material S2, Supplementary Tables S1a–S1i). Compared with nurses in Northeast China, nurses in East China had higher odds of correct responses across all nine adjusted models, with adjusted odds ratios (aORs) ranging from 1.22 for flushing fluid selection to 2.37 for NGT pre-insertion tube length measurement. Nurses in South China also had higher odds of correct responses for most items, including NGT pre-insertion tube length measurement (aOR = 3.36, 95% CI: 2.69–4.19), management when gastric aspirate could not be obtained (aOR = 3.54, 95% CI: 2.62–4.78), PP tube aspiration pH interpretation (aOR = 2.10, 95% CI: 1.72–2.57), and flushing frequency during continuous feeding (aOR = 2.68, 95% CI: 2.24–3.21). However, the regional pattern was item-specific; for flushing fluid selection, nurses in South China and Southwest China had lower odds of correct responses (aOR = 0.81, 95% CI: 0.66–0.98; and aOR = 0.58, 95% CI: 0.47–0.71, respectively).

**Table 6:** Accuracy of nurses’ knowledge regarding NGT/OGT/PP tubes by participant and institutional characteristics.

Variables *N* (%)		NGT/OGT				PP tube				
		Pre-insertion tube length measurement (NGT)	Aspiration pH	Non-aspiration Protocol	Tube tip verification timing	Pre-insertion tube length measurement (NJT)	Aspiration pH	Flushing frequency	Flushing fluid	Tube tip verification timing
Region	Northeast	130 (13.7)	698 (73.4)	64 (6.7)	322 (33.9)	244 (25.7)	219 (23.0)	367 (38.6)	262 (27.6)	226 (23.8)
	East	861 (27.9)	2,427 (78.5)	387 (12.5)	1,213 (39.2)	972 (31.4)	1,010 (32.7)	1,688 (54.6)	978 (31.6)	988 (32.0)
	Central	304 (14.7)	1,302 (63.1)	140 (6.8)	733 (35.5)	465 (22.5)	434 (21.0)	992 (48.0)	536 (26.0)	540 (26.2)
	North	228(10.8)	1,404 (66.3)	97 (4.6)	762 (36.0)	499 (23.6)	484 (22.8)	969 (45.7)	513 (24.2)	499 (23.6)
	South	447 (35.7)	1,006 (80.4)	228 (18.2)	449 (35.9)	478 (38.2)	446 (35.7)	784 (62.7)	288 (23.0)	366 (29.3)
	Northwest	143 (12.4)	615 (53.3)	61 (5.3)	402 (34.8)	248 (21.5)	192 (16.6)	473 (41.0)	344 (29.8)	260 (22.5)
	Southwest	144 (12.5)	727 (63.0)	59 (5.1)	401 (34.8)	232 (20.1)	239 (20.7)	429 (37.2)	203 (17.6)	275 (23.8)
Hospital type	CH	1880 (19.3)	6,753 (69.4)	839 (8.6)	3,514 (36.1)	2,573 (26.4)	2,473 (25.4)	4,643 (47.7)	2,617 (26.9)	2,587 (26.6)
	MCHH	105 (15.9)	404 (61.1)	45 (6.8)	234 (35.4)	136 (20.6)	132 (20.0)	297 (44.9)	156 (23.6)	140 (21.2)
	GHP	272 (19.5)	1,022 (73.3)	152 (10.9)	534 (38.3)	429 (30.8)	419 (30.1)	762 (54.7)	351 (25.2)	427 (30.6)
Department	MW	794 (15.4)	3,461 (67.1)	454 (8.8)	1740 (33.8)	1,266 (24.6)	1,208 (23.4)	2,260 (43.8)	1,458 (28.3)	1,270 (24.6)
	MSW	39 (16.7)	180 (76.9)	19 (8.1)	72 (30.8)	60 (25.6)	58 (24.8)	103 (44.0)	67 (28.6)	60 (25.6)
	SW	459 (17.0)	1893 (70.1)	234 (8.7)	1,002 (37.1)	721 (26.7)	698 (25.9)	1,274 (47.2)	762 (28.2)	743 (27.5)
	Neonatal unit	599 (27.3)	1,496 (68.3)	173 (7.9)	869 (39.7)	542 (24.7)	540 (24.7)	1,002 (45.7)	517 (23.6)	561 (25.6)
	PICU	366 (24.3)	1,149 (76.2)	156 (10.4)	599 (39.8)	549 (36.4)	520 (34.5)	1,063 (70.5)	320 (21.2)	520 (34.5)
Sex	Female	2,168 (19.0)	7,891 (69.2)	994 (8.7)	4,142 (36.3)	3,041 (26.7)	2,927 (25.7)	5,509 (48.3)	3,032 (26.6)	3,046 (26.7)
	Male	89 (23.1)	288 (74.6)	42 (10.9)	140 (36.3)	97 (25.1)	97 (25.1)	193 (50.0)	92 (23.8)	108 (28.0)
Age (yrs)	18–25	326 (23.4)	1,070 (76.8)	156 (11.2)	511 (36.7)	438 (31.4)	356 (25.5)	796 (57.1)	409 (29.3)	421 (30.2)
	26–30	541 (18.3)	2046 (69.0)	258 (8.7)	1,122 (37.9)	803 (27.1)	714 (24.1)	1,468 (49.5)	795 (26.8)	810 (27.3)
	31–35	690 (19.4)	2,399 (67.5)	292 (8.2)	1,254 (35.3)	891 (25.1)	867 (24.4)	1,646 (46.3)	882 (24.8)	897 (25.2)
	36–40	458 (18.1)	1713 (67.5)	217 (8.6)	923 (36.4)	662 (26.1)	682 (26.9)	1,212 (47.8)	693 (27.3)	697 (27.5)
	>40	242 (18.1)	951 (71.2)	113 (8.5)	472 (35.3)	344 (25.7)	405 (30.3)	580 (43.4)	345 (25.8)	329 (24.6)
Professional title	None	371 (20.3)	1,274 (69.8)	161 (8.8)	630 (34.5)	516 (28.3)	430 (23.6)	926 (50.7)	536 (29.4)	481 (26.4)
	Senior Nurse	902 (18.4)	3,297 (67.4)	418 (8.5)	1776 (36.3)	1,266 (25.9)	1,145 (23.4)	2,359 (48.2)	1,318 (27.0)	1,290 (26.4)
	Supervisor Nurse	899 (19.3)	3,273 (70.4)	409 (8.8)	1714 (36.9)	1,230 (26.5)	1,277 (27.5)	2,200 (47.3)	1,155 (24.8)	1,267 (27.3)
	Associate Chief Nurse+	85 (20.1)	335 (79.2)	48 (11.3)	162 (38.3)	126 (29.8)	172 (40.7)	217 (51.3)	115 (27.2)	116 (27.4)
Position	Clinical Nurse	2,119 (19.0)	7,697 (68.9)	964 (8.6)	4,045 (36.2)	2,960 (26.5)	2,776 (24.9)	5,391 (48.3)	2,962 (26.5)	2,982 (26.7)
	(Deputy) Head Nurse	138 (22.2)	482 (77.5)	72 (11.6)	237 (38.1)	178 (28.6)	248 (39.9)	311 (50.0)	162 (26.0)	172 (27.7)
Education	Associate Degree	245 (21.1)	798 (68.7)	117 (10.1)	397 (34.2)	336 (28.9)	248 (21.3)	573 (49.3)	320 (27.5)	288 (24.8)
	Bachelor’s Degree	1947 (18.7)	7,205 (69.2)	887 (8.5)	3,807 (36.5)	2,728 (26.2)	2,695 (25.9)	5,011 (48.1)	2,749 (26.4)	2,805 (26.9)
	Post-graduate Degree	65 (31.4)	176 (85.0)	32 (15.5)	78 (37.7)	74 (35.7)	81 (39.1)	118 (57.0)	55 (26.6)	61 (29.5)
Pediatric working experience (yrs)	1 ~ 5	580 (20.2)	2078 (72.5)	281 (9.8)	1,051 (36.7)	848 (29.6)	719 (25.1)	1,539 (53.7)	809 (28.2)	832 (29.0)
	6 ~ 10	675 (19.3)	2,422 (69.1)	310 (8.8)	1,293 (36.9)	926 (26.4)	863 (24.6)	1,674 (47.8)	911 (26.0)	905 (25.8)
	11 ~ 20	829 (18.2)	3,052 (67.0)	364 (8.0)	1,641 (36.0)	1,152 (25.3)	1,189 (26.1)	2,132 (46.8)	1,188 (26.1)	1,220 (26.8)
	>20	173 (20.1)	627 (72.9)	81 (9.4)	297 (34.5)	212 (24.7)	253 (29.4)	357 (41.5)	216 (25.1)	197 (22.9)

CH, Children’s hospitals; MCHH, Maternal and child health hospitals; GHP, General hospitals with pediatric departments; MW, Medical ward; MSW, Medical and surgical ward; SW, Surgical ward; NGT, Nasogastric tube; OGT, Orogastric tube; PP tube, post-pyloric tube. + Includes Associate Chief Nurse and Chief Nurse.

Hospital type and ward type also remained associated with selected knowledge items after adjustment. Compared with children’s hospitals, general hospitals with pediatric departments were associated with higher odds of correct responses for six items, including NGT/OGT aspiration pH interpretation, management when gastric aspirate could not be obtained, NJT pre-insertion tube length measurement, PP tube aspiration pH interpretation, flushing frequency during continuous feeding, and PP tube-tip verification timing, with aORs ranging from 1.34 to 1.73. Compared with medical wards, intensive care units were associated with higher odds of correct responses for several technical and maintenance-related items, particularly flushing frequency during continuous feeding (aOR = 2.89, 95% CI: 2.55–3.28). Nevertheless, intensive care units showed lower odds of correct responses for flushing fluid selection (aOR = 0.68, 95% CI: 0.59–0.78), indicating that ward-level differences were not uniform across all knowledge domains.

Nurse-level characteristics were associated with correct responses to some knowledge items; however, the associations varied across items. Compared with clinical nurses, head or deputy head nurses had higher odds of correct responses for NGT pre-insertion tube length measurement (aOR = 1.28, 95% CI: 1.02–1.61), management when gastric aspirate could not be obtained (aOR = 1.41, 95% CI: 1.05–1.89), and PP tube aspiration pH interpretation (aOR = 1.57, 95% CI: 1.28–1.92). Postgraduate education was associated with higher odds of correct responses for NGT pre-insertion tube length measurement (aOR = 1.71, 95% CI: 1.20–2.45), NGT/OGT aspiration pH interpretation (aOR = 2.20, 95% CI: 1.44–3.36), PP tube aspiration pH interpretation (aOR = 1.52, 95% CI: 1.08–2.14), and flushing frequency during continuous feeding (aOR = 1.38, 95% CI: 1.01–1.90). Longer pediatric nursing experience was not consistently associated with higher knowledge accuracy; nurses with more than 20 years of pediatric experience had lower odds of correct responses for PP tube aspiration pH interpretation (aOR = 0.68, 95% CI: 0.49–0.94) and flushing frequency during continuous feeding (aOR = 0.73, 95% CI: 0.55–0.96). The full crude and adjusted logistic regression results are presented in Electronic Supplementary Material S2, Supplementary Tables S1a–S1i.

## 4 Discussion

The aim of this study was to estimate the point prevalence of enteral feeding tube use among hospitalized children in China and to evaluate nurses’ current knowledge of tube management. The prevalence of enteral feeding tubes in China was 7.7% during the survey period, which is lower than the 24% (1,995/8,333) reported in the NOVEL Project in the United States (7). This difference should be interpreted cautiously because the two studies differed in country context, hospital composition, patient case mix, and sampling approach. In our study, enteral feeding tube use was most common in PICUs and neonatal units, which may be related to the high proportion of preterm infants and critically ill children in these settings (3, 4). These children are often unable to take sufficient nutrients orally while having high nutritional needs, making enteral tube feeding clinically necessary.

Gastric tubes constituted the predominant modality in our study, offering advantages in cyclic feeding regimens due to better alignment with physiological characteristics (12). Notably, OGT (54.9%) surpassed NGT utilization (41.1%), contrasting with the findings from the NOVEL study in the United States (NGT 66% vs. OGT 21%) (7). This discrepancy may reflect the high proportion of neonates (64.6%) in our study, as nasal pathway occupation by oxygen therapy or respiratory support devices frequently precludes NGT placement in this population (13). While OGT access addresses nutritional requirements in such cases, it presents challenges including insecure fixation risks (malposition, dislodgement) and potential interference with oral motor skill development critical for transitioning to oral feeding.

Post-pyloric tubes were utilized in 3.2% of cases, substantially lower than the 13% reported in the USA NOVEL study (7). Among clinical departments, the PICU had the highest utilization rate of PP tubes. This discrepancy may reflect differences in case mix or clinical awareness regarding enteral nutrition indications. Current guidelines explicitly recommend PP feeding for high-risk populations including those with aspiration predisposition or gastroparesis, as this approach mitigates gastric retention and reflux-related complications while facilitating nutritional goal attainment (9, 10). Post-pyloric tube placement has a certain technical threshold, requiring a clinician to undergo specialized training (14, 15).

Gastrostomy, jejunostomy, and PEG-J tubes collectively accounted for only 1.3% of devices in our study. Limited directly comparable international point-prevalence data are currently available. Given the risks associated with prolonged nasoenteric feeding, including nasal mucosal injury, reflux esophagitis, and otitis media, clinical guidelines recommend transitioning to gastrostomy when enteral nutrition is expected to exceed 4 weeks to enhance feeding safety, reduce social stigma, and improve quality of life (16). However, the low observed use of gastrostomy and jejunostomy tubes may reflect multiple factors, including patient and caregiver preferences, clinicians’ familiarity with long-term enteral access options, multidisciplinary communication, and local practice patterns. A survey of clinicians in China found that NGT feeding was the predominant enteral nutrition method, whereas PEG was used less frequently, and identified insufficient clinician knowledge of PEG, resistance from patients and families, and poor interdepartmental cooperation as potential barriers to PEG use (17). Other evidence has similarly suggested that insufficient knowledge of PEG feeding and inadequate multidisciplinary communication may contribute to its underuse (18). In addition, studies from Asian settings have suggested that acceptance of NGT feeding may be influenced by patients’ and caregivers’ previous experiences, cultural and religious beliefs, concerns about additional surgical procedures, and the perceived importance of maintaining body integrity in patients who already have underlying disease or disability (19). Implementation strategies should therefore prioritize improved informational and technical support for patients requiring long-term tube feeding and their caregivers.

Inappropriate feeding tube placement length may lead to dumping syndrome during bolus feedings when excessively long, while overly short tube insertion length risks esophageal placement and increased aspiration risk. Our data showed that most nurses used the HX method to estimate gastric tube insertion length. The predominance of HX and the Nasal tip → Earlobe → Xiphoid (NEX) methods aligns with their explicit endorsement in Chinese foundational nursing textbooks (20), despite evidence demonstrating the superior accuracy of the Nasal tip → Earlobe → Midpoint from xiphoid to umbilicus (NEMU) method in reducing aspiration incidence and enhancing care safety (11). This discrepancy may reflect a persistent gap between emerging evidence and routine clinical practice. For PP tube placement, standardized measurement protocols for tube insertion depth remain undefined. Current clinical practice predominantly employs NEMU → left/right costal margin at midaxillary line +5–10 cm. However, this approach proves inadequate for ensuring accurate placement depth across pediatric populations with substantial age and height variations. Alternative methods including NEMU → Left/right iliac crests and NEX → Left/right iliac crests similarly exhibit limited pediatric adaptability. Future research should prioritize developing individualized measurement protocols incorporating anatomical characteristics and growth parameters for enhanced precision.

Accurate verification of feeding tube tip position is a prerequisite for safe enteral administration. Administration of liquids, nutrients, or pharmaceuticals should be withheld until proper placement is confirmed (4), although 0.2% of nurses reported instilling water for verification when unable to aspirate contents. Auscultation has inadequate specificity for differentiating gastric versus pulmonary placement and should not be used as the sole confirmation method (21). Our survey data showed that aspiration, auscultation, and bubble observation remained predominant verification methods. The coexistence of traditional methods with safer evidence-based approaches may reflect incomplete translation of updated evidence-based recommendations into routine nursing practice. These traditional methods may persist because they have long been embedded in nursing education, textbooks, and ward routines. Regular curricular updates and explicit warnings regarding controversial or outdated techniques may help support the transition toward more evidence-based tube verification practices. Radiographic confirmation remains the reference standard for blindly inserted feeding tubes, but its limitations include radiation exposure and potential delays in feeding initiation. Therefore, radiography may not always be the preferred routine practice for NGT or OGT placement confirmation. When radiography is unavailable, combined assessment using aspirate characteristics, pH testing, and external length measurement is advised, with more than two concordant methods recommended for confirmation (4).

Contemporary advancements in enteral access techniques have expanded beyond traditional blind insertion to include endoscopic guidance, electromagnetic navigation, and point-of-care ultrasound (22). Nurses can enhance procedural success and professional value in pediatric cases by using auxiliary medical devices. Bedside ultrasound enhances placement accuracy and safety (23, 24), with 55.9% utilization reported among nurses experienced in PP tube insertion in our study. Electromagnetic navigation systems enable real-time tracking with superior sensitivity and specificity, reducing radiographic confirmation needs and radiation burden (25–27), though constrained by higher costs and tube diameters that are inappropriate for smaller children and infants. Clinical implementation should prioritize patient-centered outcomes through strategic selection of insertion modalities, balancing technical characteristics, resource availability, and cost-effectiveness to optimize safety and procedural success.

After adjustment, nurses’ knowledge gaps showed item-specific patterns across regions, hospital types, departments, and nurse-level characteristics. At the regional level, nurses in East and South China had higher adjusted odds of correct responses for most knowledge items. At the institutional level, nurses in general hospitals with pediatric departments had higher odds of correct responses than nurses in children’s hospitals for several items after adjustment. At the departmental level, PICU nurses had higher odds of correct responses for several procedure-related knowledge items, including tube placement, tube-tip verification, and flushing frequency. These associations may partly reflect differences in clinical case exposure, local training resources, institutional nursing protocols, and the priority given to enteral feeding tube management; however, because these explanatory factors were not directly measured, these interpretations should be considered hypothesis-generating. Moreover, the knowledge advantages observed in South China and PICUs did not extend to fundamental maintenance items, such as flushing solution selection. This pattern suggests that proficiency in procedural knowledge does not necessarily translate into parallel proficiency in routine maintenance knowledge. Current guidelines for enteral nutrition and pediatric tube feeding emphasize not only initial placement and tip verification but also safe use and ongoing maintenance, including standardized flushing protocols (28, 29). These adjusted findings therefore support targeted education based on setting-level knowledge distribution and item-specific deficits rather than reliance on aggregated correct response rates alone.

At the individual level, higher educational background and managerial position were associated with correct responses to selected items, whereas longer pediatric nursing experience was not consistently associated with higher knowledge accuracy. This finding suggests that accumulated clinical experience alone may not ensure that nurses remain up to date with current evidence-based recommendations. Barriers to evidence-based nursing practice have been reported in previous studies and may include established ward routines, limited access to updated evidence, and insufficient structured education (30). Therefore, future continuing education programs should be stratified not only by geographic region and clinical setting but also by professional career stage. Experienced nurses and ward nurse managers should be included in periodic knowledge-updating systems because they influence local nursing routines, informal preceptorship practices, and the translation of updated tube-feeding guidelines across clinical teams.

## 5 Limitations

Several limitations should be acknowledged. First, the cross-sectional point-prevalence design captured only a snapshot of clinical status and nurses’ knowledge, precluding causal inference between institutional characteristics and nurses’ knowledge. Selection bias may exist because hospitals were recruited voluntarily through professional networks, potentially over representing institutions with greater expertise or interest in enteral nutrition; thus, caution is warranted when generalizing these findings nationwide. Second, despite standardized training, data heterogeneity remains unavoidable due to variations in local information systems and coordinators’ experience across the 52 hospitals. Additionally, the self-reported questionnaire may be subject to recall and social desirability biases, and the lack of direct observation means that these findings reflect knowledge distribution rather than actual bedside performance. Third, we did not collect data on nurses’ prior training, institutional protocols, detailed tube-feeding indications, disease severity, nutritional risk, patient-level combinations of verification methods, or tube-related adverse events; therefore, these factors could not be adjusted for in the regression models or used to evaluate the safety impact of different verification practices. Future studies should integrate practical competency evaluations, institutional protocol surveys, detailed patient-level clinical variables, and clinical outcome data to comprehensively assess the quality and safety of pediatric feeding tube management.

## 6 Conclusion

This nationwide multicenter study describes the current use of enteral feeding tubes in hospitalized children in China. Although enteral feeding tube placement is common, substantial gaps remain in nurses’ knowledge compared with current evidence, particularly in tube length estimation and placement verification. Adjusted analyses further indicated that these knowledge deficits were heterogeneous and item-specific, differing by region, hospital type, department type, and individual nurse attributes. These findings emphasize the need for standardized protocols and targeted evidence-based training to improve the safety and quality of enteral nutrition care in children.

## Data Availability

The raw data supporting the conclusions of this article will be made available by the authors, without undue reservation.
