# Supplementary material for: Enteral feeding tube use among pediatric patients and nurses’ knowledge in China: a 52-hospital point-prevalence and cross-sectional survey study
Source: Front Nutr. 2026 Aug 18;13:1854191. doi: 10.3389/fnut.2026.1854191 (PMC13529481; doi:10.3389/fnut.2026.1854191)
Supplement: Supplementary file 1 [file Data_sheet_1.docx]

**Electronic Supplementary Material 1 - English Version of the Survey**

**Survey 1:  *Point-prevalence data collection form***

**Section 1: Patient demographics**

1. Institution: ____

2. Province and city: ____

3. Number of hospitalized children: ____

4. Gender: □Male □Female

5. Date of Birth: ____

6. Department:____

7. Department category:

□Neonatal unit □ Medical Ward □Surgical Ward □Pediatric Intensive Care Unit □Medical and Surgical Ward

8. Height (cm): ____

9. Weight (kg): ____

10. Primary diagnosis:____

**Section 2: Feeding tube characteristics**

1. Which type of feeding tube was inserted in the child?

□Nasogastric tube □Orogastric tube □Post-pyloric tube □Gastrostomy tube □Jejunostomy tube □Percutaneous endoscopic gastrostomy with jejunal extension

**Section 3: Department information**

1. Institution: ____

2. Department:____

3. Department category:____

4. Total hospitalized children:____

**Survey 2: *Survey of pediatric nurses’ knowledge and experience with enteral feeding tubes***

**Section 1: General characteristics**

1. Institution: ____
2. Province and city: ____

3. Ward:____

4. Ward category:

□Neonatal unit □ Medical Ward □Surgical Ward □Pediatric Intensive Care Unit □Medical and Surgical Ward

5. Gender: □Male □Female

6. Age: ____

7. Professional title: □None □Senior Nurse □Supervisor Nurse □Associate Chief Nurse+

8. Education level: □Associate Degree □Bachelor's degree □Post-graduate Degree

9. Position: □Clinical Nurse □(Deputy) Head Nurse

10. Pediatric working experience (yrs): ____

**Section 2: Nurses’ knowledge and experience of enteral feeding tube**

1. Have you ever placed a gastric tube for children? □Yes □No
2. Which method do you (think is appropriate to) use to measure the insertion length before placing a Nasogastric tube in children?

□HX

□NEX

□NEMU

□Others

□Not clear

3. Which of the following methods do you (think should) be used to confirm the position of the gastric tube tip? (Select all that apply)

□Aspiration

□Visual inspection of aspiration color

□pH testing of aspiration

□Auscultation

□Bubble observation

□X-ray

□Ultrasound

□Endoscope

□Others

4. What pH value indicates that the gastric tube is correctly positioned in the stomach?

□ ≤ 5.5

□5.6—10

□>10

□Not clear

5. If gastric contents cannot be aspirated, what would you (do / consider appropriate to do)? (Select all that apply)

□Positional adjustment with respiration

□Air insufflation

□X-ray

□Withdrawal and replacement

□Not clear

□Others

6. At which of the following times do you (think it is necessary to) re-verify the position of the gastric tube tip? (except dislodgement suspicion) (Select all that apply)

□Pre-feeding

□Per shift

□Everyday

□Every week

□Placement confirmed, no further checks

□Others

□Not clear

7. Have you ever inserted a post-pyloric tube in children? □Yes □No
8. Which method do you (think is appropriate to) use to measure the insertion length before placing a Nasojejunal tube?

□NEX→Left/right iliac crest

□NEMU→Left/right iliac crest

□NEX→Left/right costal margin

□NEMU→Left/right costal margin-midaxillary line intersection +5-10 cm

□Not clear

□Others

9. Which methods do you (think are appropriate) for confirming the position of the post-pyloric tube tip? (Select all that apply)

□Aspiration

□pH testing of aspiration

□Visual inspection of aspiration color

□Auscultation

□Aspirate volume < Infuse volume

□X-ray

□Ultrasound

□Endoscope

□Others

□Not clear

10. What pH value indicates that the Post-pyloric tube is correctly positioned in the intestine?

□≤5.5

□5.6-7

□>7

□Not clear

11. What do you (think is the appropriate) flushing frequency during continuous enteral feeding?

□Every 4-6 h

□Every 12h

□Every day

□Every week

□Not clear

□Others

12. What type of solution do you (think should) be used to flush the post-pyloric tube? (Select all that apply)

□Tap water

□Warm boiled water

□0.9% NaCl

□Sterile water for injection

□Not clear

□Others

13. At which of the following times do you (think it is necessary) to re-verify the position of the Post-pyloric tube tip? (except dislodgement suspicion) (Select all that apply)

□Pre-feeding

□Per shift

□Every day

□Every week

□Placement confirmed, no further checks

□Others

□Not clear

14. What devices have you used to assist with nasojejunal tube insertion? (Select all that apply)

□Bedside ultrasound

□Endoscopy

□Electromagnetic guidance

□Blind insertions

□X-ray

15. What devices have you used to assist with nasojejunal tube tip localization? (Select all that apply)

□X-ray

□Bedside ultrasound

□Endoscopy

□Electromagnetic guidance

□Auscultation
