# Supplementary material for: Enteral feeding tube use among pediatric patients and nurses’ knowledge in China: a 52-hospital point-prevalence and cross-sectional survey study
Source: Front Nutr. 2026 Aug 18;13:1854191. doi: 10.3389/fnut.2026.1854191 (PMC13529481; doi:10.3389/fnut.2026.1854191)
Supplement: Supplementary file 2 [file Table_1.docx]

**Electronic Supplementary Material 2 - Electronic Supplementary Table**

**Table S1a. Univariable and multivariable logistic regression analyses of factors associated with correct responses for pre-insertion tube length measurement for NGT**

| Variable | Univariable analysis | | | | | Multivariable analysis | | | | |
| --- | --- | --- | --- | --- | --- | --- | --- | --- | --- | --- |
|  | β | SE | wald χ² | P | OR (95% CI) | β | SE | wald χ² | P / omnibus | aOR (95% CI) |
| **Region** |  |  |  | **<.001** |  |  |  |  | **<.001** |  |
| Northeast |  |  |  |  | 1.00 (Reference) |  |  |  |  | 1.00 (Reference) |
| North | -0.273 | 0.118 | 5.372 | 0.02 | 0.76 (0.61~0.96) | -0.295 | 0.119 | 6.183 | **0.013** | **0.75 (0.59~0.94)** |
| East | 0.891 | 0.103 | 75.443 | <.001 | 2.44 (1.99~2.98) | 0.864 | 0.104 | 68.935 | **<.001** | **2.37 (1.94~2.91)** |
| South | 1.256 | 0.111 | 127.3 | <.001 | 3.51 (2.82~4.37) | 1.212 | 0.113 | 114.556 | **<.001** | **3.36 (2.69~4.19)** |
| Central | 0.086 | 0.113 | 0.584 | 0.445 | 1.09 (0.87~1.36) | 0.048 | 0.114 | 0.178 | 0.673 | 1.05 (0.84~1.31) |
| Northwest | -0.114 | 0.13 | 0.767 | 0.381 | 0.89 (0.69~1.15) | -0.065 | 0.131 | 0.249 | 0.618 | 0.94 (0.73~1.21) |
| Southwest | -0.105 | 0.13 | 0.653 | 0.419 | 0.9 (0.7~1.16) | -0.141 | 0.131 | 1.158 | 0.282 | 0.87 (0.67~1.12) |
| **Hospital type** |  |  |  | 0.09 |  |  |  |  |  |  |
| CH |  |  |  |  | 1.00 (Reference) |  |  |  |  |  |
| MCHH | -0.237 | 0.109 | 4.7 | 0.03 | 0.79 (0.64~0.98) |  |  |  |  |  |
| GHP | 0.012 | 0.072 | 0.03 | 0.863 | 1.01 (0.88~1.17) |  |  |  |  |  |
| **Ward** |  |  |  | **<.001** |  |  |  |  |  |  |
| MW |  |  |  |  | 1.00 (Reference) |  |  |  |  | 1.00 (Reference) |
| MSW | 0.094 | 0.18 | 0.275 | 0.6 | 1.1 (0.77~1.56) | -0.072 | 0.184 | 0.153 | 0.696 | 0.93 (0.65~1.33) |
| SW | 0.118 | 0.064 | 3.409 | 0.065 | 1.13 (0.99~1.28) | 0.055 | 0.066 | 0.707 | 0.401 | 1.06 (0.93~1.2) |
| Neonatal unit | 0.726 | 0.062 | 139.252 | <.001 | 2.07 (1.83~2.33) | 0.704 | 0.064 | 122.164 | **<.001** | **2.02 (1.79~2.29)** |
| PICU | 0.567 | 0.071 | 62.975 | <.001 | 1.76 (1.53~2.03) | 0.505 | 0.074 | 46 | **<.001** | **1.66 (1.43~1.92)** |
| **Sex** |  |  |  |  |  |  |  |  |  |  |
| Male |  |  |  |  | 1.00 (Reference) |  |  |  |  | 1.00 (Reference) |
| Female | 0.244 | 0.123 | 3.92 | 0.048 | 1.28 (1~1.63) | 0.073 | 0.131 | 0.317 | 0.574 | 1.08 (0.83~1.39) |
| **Age（yrs）** |  |  |  | **<.001** |  |  |  |  |  |  |
| 18-25 |  |  |  |  | 1.00 (Reference) |  |  |  |  | 1.00 (Reference) |
| 26-30 | -0.313 | 0.079 | 15.607 | <.001 | 0.73 (0.63~0.85) | -0.254 | 0.086 | 8.688 | **0.003** | **0.78 (0.66~0.92)** |
| 31-35 | -0.237 | 0.076 | 9.708 | 0.002 | 0.79 (0.68~0.92) | -0.09 | 0.085 | 1.118 | 0.29 | 0.91 (0.77~1.08) |
| 36-40 | -0.326 | 0.082 | 15.95 | <.001 | 0.72 (0.62~0.85) | -0.122 | 0.091 | 1.785 | 0.182 | 0.89 (0.74~1.06) |
| >40 | -0.322 | 0.095 | 11.458 | <.001 | 0.73 (0.6~0.87) | -0.177 | 0.107 | 2.73 | 0.098 | 0.84 (0.68~1.03) |
| **Professional title** |  |  |  | 0.314 |  |  |  |  |  |  |
| None |  |  |  |  | 1.00 (Reference) |  |  |  |  |  |
| Senior Nurse | -0.121 | 0.069 | 3.065 | 0.08 | 0.89 (0.78~1.02) |  |  |  |  |  |
| Supervisor Nurse | -0.062 | 0.069 | 0.817 | 0.366 | 0.94 (0.82~1.08) |  |  |  |  |  |
| Associate Chief Nurse+ | -0.015 | 0.135 | 0.012 | 0.914 | 0.99 (0.76~1.28) |  |  |  |  |  |
| **Position** |  |  |  |  |  |  |  |  |  |  |
| Clinical Nurse |  |  |  |  | 1.00 (Reference) |  |  |  |  | 1.00 (Reference) |
| (Deputy) Head Nurse | 0.197 | 0.099 | 3.904 | 0.048 | 1.22 (1~1.48) | 0.247 | 0.115 | 4.6 | **0.032** | **1.28 (1.02~1.61)** |
| **Education** |  |  |  | <.001 |  |  |  |  |  |  |
| Associate Degree |  |  |  |  | 1.00 (Reference) |  |  |  |  | 1.00 (Reference) |
| Bachelor's Degree | -0.151 | 0.076 | 3.903 | 0.048 | 0.86 (0.74~1) | -0.007 | 0.085 | 0.007 | 0.936 | 0.99 (0.84~1.17) |
| Post-graduate Degree | 0.538 | 0.166 | 10.503 | 0.001 | 1.71 (1.24~2.37) | 0.539 | 0.182 | 8.738 | **0.003** | **1.71 (1.2~2.45)** |
| **Pediatric working experience（yrs）** | | | | 0.143 |  |  |  |  |  |  |
| 1~5 |  |  |  |  | 1.00 (Reference) |  |  |  |  |  |
| 6~10 | -0.061 | 0.063 | 0.944 | 0.331 | 0.94 (0.83~1.06) |  |  |  |  |  |
| 11~20 | -0.132 | 0.06 | 4.783 | 0.029 | 0.88 (0.78~0.99) |  |  |  |  |  |
| >20 | -0.008 | 0.097 | 0.006 | 0.938 | 0.99 (0.82~1.2) |  |  |  |  |  |

CH = Children’s hospitals; MCHH = Maternal and child health hospitals; GHP = General hospitals with pediatric departments; MW, Medical ward; MSW, Medical and surgical ward; SW, Surgical ward; PICU, Pediatric intensive care unit.

**Table S1b. Univariable and multivariable logistic regression analyses of factors associated with correct responses for NGT/OGT aspiration pH interpretation**

| Variable | Univariable analysis | | | | | Multivariable analysis | | | | |
| --- | --- | --- | --- | --- | --- | --- | --- | --- | --- | --- |
|  | β | SE | Wald χ² | P | OR (95% CI) | β | SE | wald χ² | P / omnibus | aOR (95% CI) |
| **Region** |  |  |  | <.001 |  |  |  |  | **<.001** |  |
| Northeast |  |  |  |  | 1.00 (Reference) |  |  |  |  | 1.00 (Reference) |
| North | -0.34 | 0.087 | 15.424 | <.001 | 0.71 (0.6~0.84) | -0.312 | 0.089 | 12.411 | **<.001** | **0.73 (0.62~0.87)** |
| East | 0.28 | 0.085 | 10.722 | 0.001 | 1.32 (1.12~1.56) | 0.288 | 0.088 | 10.563 | **0.001** | **1.33 (1.12~1.59)** |
| South | 0.398 | 0.102 | 15.116 | <.001 | 1.49 (1.22~1.82) | 0.36 | 0.106 | 11.547 | **<.001** | **1.43 (1.17~1.76)** |
| Central | -0.48 | 0.086 | 30.924 | <.001 | 0.62 (0.52~0.73) | -0.531 | 0.089 | 35.992 | **<.001** | **0.59 (0.49~0.7)** |
| Northwest | -0.885 | 0.094 | 88.325 | <.001 | 0.41 (0.34~0.5) | -0.828 | 0.096 | 74.053 | **<.001** | **0.44 (0.36~0.53)** |
| Southwest | -0.483 | 0.095 | 25.595 | <.001 | 0.62 (0.51~0.74) | -0.425 | 0.1 | 18.174 | **<.001** | **0.65 (0.54~0.8)** |
| **Hospital type** |  |  |  | <.001 |  |  |  |  |  |  |
| CH |  |  |  |  | 1.00 (Reference) |  |  |  |  | 1.00 (Reference) |
| MCHH | -0.192 | 0.064 | 8.903 | 0.003 | 0.83 (0.73~0.94) | -0.284 | 0.089 | 10.2 | **0.001** | **0.75 (0.63~0.9)** |
| GHP | -0.558 | 0.1 | 31.066 | <.001 | 0.57 (0.47~0.7) | 0.326 | 0.068 | 22.814 | **<.001** | **1.39 (1.21~1.58)** |
| **Ward** |  |  |  | <.001 |  |  |  |  |  |  |
| MW |  |  |  |  | 1.00 (Reference) |  |  |  |  | 1.00 (Reference) |
| MSW | 0.49 | 0.158 | 9.626 | 0.002 | 1.63 (1.2~2.23) | 0.464 | 0.162 | 8.175 | **0.004** | **1.59 (1.16~2.19)** |
| SW | 0.14 | 0.051 | 7.398 | 0.007 | 1.15 (1.04~1.27) | 0.121 | 0.053 | 5.187 | **0.023** | **1.13 (1.02~1.25)** |
| Neonatal unit | 0.053 | 0.055 | 0.932 | 0.334 | 1.05 (0.95~1.17) | 0.007 | 0.057 | 0.014 | 0.907 | 1.01 (0.9~1.13) |
| PICU | 0.452 | 0.067 | 45.024 | <.001 | 1.57 (1.38~1.79) | 0.38 | 0.07 | 29.675 | **<.001** | **1.46 (1.28~1.68)** |
| **Sex** |  |  |  |  |  |  |  |  |  |  |
| Male |  |  |  |  | 1.00 (Reference) |  |  |  |  | 1.00 (Reference) |
| Female | 0.268 | 0.119 | 5.094 | 0.024 | 1.31 (1.04~1.65) | 0.106 | 0.124 | 0.72 | 0.396 | 1.11 (0.87~1.42) |
| **Age（yrs）** |  |  |  | <.001 |  |  |  |  |  |  |
| 18-25 |  |  |  |  | 1.00 (Reference) |  |  |  |  | 1.00 (Reference) |
| 26-30 | -0.393 | 0.075 | 27.616 | <.001 | 0.68 (0.58~0.78) | -0.483 | 0.098 | 24.179 | **<.001** | **0.62 (0.51~0.75)** |
| 31-35 | -0.465 | 0.073 | 40.859 | <.001 | 0.63 (0.54~0.72) | -0.593 | 0.12 | 24.603 | **<.001** | **0.55 (0.44~0.7)** |
| 36-40 | -0.463 | 0.076 | 36.818 | <.001 | 0.63 (0.54~0.73) | -0.564 | 0.134 | 17.802 | **<.001** | **0.57 (0.44~0.74)** |
| >40 | -0.29 | 0.088 | 10.995 | <.001 | 0.75 (0.63~0.89) | -0.598 | 0.16 | 13.949 | **<.001** | **0.55 (0.4~0.75)** |
| **Professional title** |  |  |  | <.001 |  |  |  |  |  |  |
| None |  |  |  |  | 1.00 (Reference) |  |  |  |  | 1.00 (Reference) |
| Senior Nurse | -0.111 | 0.059 | 3.476 | 0.062 | 0.9 (0.8~1.01) | 0.17 | 0.078 | 4.672 | **0.031** | **1.19 (1.02~1.38)** |
| Supervisor Nurse | 0.028 | 0.06 | 0.221 | 0.638 | 1.03 (0.91~1.16) | 0.468 | 0.089 | 27.563 | **<.001** | **1.6 (1.34~1.9)** |
| Associate Chief Nurse+ | 0.499 | 0.13 | 14.669 | <.001 | 1.65 (1.28~2.13) | 0.668 | 0.17 | 15.468 | **<.001** | **1.95 (1.4~2.72)** |
| **Position** |  |  |  |  |  |  |  |  |  |  |
| Clinical Nurse |  |  |  |  | 1.00 (Reference) |  |  |  |  | 1.00 (Reference) |
| (Deputy) Head Nurse | 0.439 | 0.098 | 20.005 | <.001 | 1.55 (1.28~1.88) | 0.216 | 0.116 | 3.499 | 0.061 | 1.24 (0.99~1.56) |
| **Education** |  |  |  | <.001 |  |  |  |  |  |  |
| Associate Degree |  |  |  |  | 1.00 (Reference) |  |  |  |  | 1.00 (Reference) |
| Bachelor's Degree | 0.023 | 0.067 | 0.115 | 0.735 | 1.02 (0.9~1.17) | 0.108 | 0.076 | 1.999 | 0.157 | 1.11 (0.96~1.29) |
| Post-graduate Degree | 0.952 | 0.205 | 21.589 | <.001 | 2.59 (1.73~3.87) | 0.79 | 0.216 | 13.381 | **<.001** | **2.2 (1.44~3.36)** |
| **Pediatric working experience（yrs）** | | | | <.001 |  |  |  |  |  |  |
| 1~5 |  |  |  |  | 1.00 (Reference) |  |  |  |  | 1.00 (Reference) |
| 6~10 | -0.164 | 0.056 | 8.699 | 0.003 | 0.85 (0.76~0.95) | -0.025 | 0.078 | 0.105 | 0.746 | 0.98 (0.84~1.14) |
| 11~20 | -0.263 | 0.052 | 25.157 | <.001 | 0.77 (0.69~0.85) | -0.19 | 0.099 | 3.679 | 0.055 | 0.83 (0.68~1) |
| >20 | 0.02 | 0.087 | 0.054 | 0.817 | 1.02 (0.86~1.21) | -0.058 | 0.159 | 0.133 | 0.716 | 0.94 (0.69~1.29) |

CH = Children’s hospitals; MCHH = Maternal and child health hospitals; GHP = General hospitals with pediatric departments; MW, Medical ward; MSW, Medical and surgical ward; SW, Surgical ward; PICU, Pediatric intensive care unit.

**Table S1c. Univariable and multivariable logistic regression analyses of factors associated with correct responses for protocol when content cannot be aspirated for NGT/OGT**

| Variable | Univariable analysis | | | | | Multivariable analysis | | | | |
| --- | --- | --- | --- | --- | --- | --- | --- | --- | --- | --- |
|  | β | SE | wald χ² | P | OR (95% CI) | β | SE | wald χ² | P / omnibus | aOR (95% CI) |
| **Region** |  |  |  | <.001 |  |  |  |  | **<.001** |  |
| Northeast |  |  |  |  | 1.00 (Reference) |  |  |  |  | 1.00 (Reference) |
| North | -0.408 | 0.166 | 6.046 | 0.014 | 0.67 (0.48~0.92) | -0.317 | 0.168 | 3.554 | 0.059 | 0.73 (0.52~1.01) |
| East | 0.685 | 0.14 | 23.778 | <.001 | 1.98 (1.51~2.61) | 0.821 | 0.145 | 32.032 | **<.001** | **2.27 (1.71~3.02)** |
| South | 1.128 | 0.149 | 57.514 | <.001 | 3.09 (2.31~4.13) | 1.263 | 0.153 | 67.937 | **<.001** | **3.54 (2.62~4.78)** |
| Central | 0.008 | 0.156 | 0.003 | 0.96 | 1.01 (0.74~1.37) | 0.036 | 0.157 | 0.053 | 0.818 | 1.04 (0.76~1.41) |
| Northwest | -0.258 | 0.185 | 1.951 | 0.163 | 0.77 (0.54~1.11) | -0.141 | 0.186 | 0.571 | 0.45 | 0.87 (0.6~1.25) |
| Southwest | -0.292 | 0.186 | 2.463 | 0.117 | 0.75 (0.52~1.08) | -0.114 | 0.19 | 0.361 | 0.548 | 0.89 (0.62~1.3) |
| **Hospital type** |  |  |  | 0.004 |  |  |  |  |  |  |
| CH |  |  |  |  | 1.00 (Reference) |  |  |  |  | 1.00 (Reference) |
| MCHH | -0.256 | 0.159 | 2.601 | 0.107 | 0.77 (0.57~1.06) | -0.214 | 0.165 | 1.674 | 0.196 | 0.81 (0.58~1.12) |
| GHP | 0.26 | 0.093 | 7.793 | 0.005 | 1.3 (1.08~1.56) | 0.551 | 0.1 | 30.277 | **<.001** | **1.73 (1.43~2.11)** |
| **Ward** |  |  |  | 0.14 |  |  |  |  |  |  |
| MW |  |  |  |  | 1.00 (Reference) |  |  |  |  |  |
| MSW | -0.089 | 0.244 | 0.131 | 0.717 | 0.92 (0.57~1.48) |  |  |  |  |  |
| SW | -0.017 | 0.084 | 0.041 | 0.84 | 0.98 (0.83~1.16) |  |  |  |  |  |
| Neonatal unit | -0.119 | 0.093 | 1.627 | 0.202 | 0.89 (0.74~1.07) |  |  |  |  |  |
| PICU | 0.179 | 0.098 | 3.346 | 0.067 | 1.2 (0.99~1.45) |  |  |  |  |  |
| **Sex** |  |  |  |  |  |  |  |  |  |  |
| Male |  |  |  |  | 1.00 (Reference) |  |  |  |  |  |
| Female | 0.246 | 0.167 | 2.167 | 0.141 | 1.28 (0.92~1.77) |  |  |  |  |  |
| **Age（yrs）** |  |  |  | 0.019 |  |  |  |  |  |  |
| 18-25 |  |  |  |  | 1.00 (Reference) |  |  |  |  | 1.00 (Reference) |
| 26-30 | -0.279 | 0.107 | 6.784 | 0.009 | 0.76 (0.61~0.93) | -0.145 | 0.114 | 1.619 | 0.203 | 0.87 (0.69~1.08) |
| 31-35 | -0.343 | 0.105 | 10.717 | 0.001 | 0.71 (0.58~0.87) | -0.18 | 0.114 | 2.511 | 0.113 | 0.84 (0.67~1.04) |
| 36-40 | -0.298 | 0.111 | 7.246 | 0.007 | 0.74 (0.6~0.92) | -0.092 | 0.121 | 0.572 | 0.45 | 0.91 (0.72~1.16) |
| >40 | -0.31 | 0.13 | 5.702 | 0.017 | 0.73 (0.57~0.95) | -0.21 | 0.144 | 2.137 | 0.144 | 0.81 (0.61~1.07) |
| **Professional title** |  |  |  | 0.286 |  |  |  |  |  |  |
| None |  |  |  |  | 1.00 (Reference) |  |  |  |  |  |
| Senior Nurse | -0.035 | 0.097 | 0.126 | 0.722 | 0.97 (0.8~1.17) |  |  |  |  |  |
| Supervisor Nurse | -0.003 | 0.097 | 0.001 | 0.975 | 1 (0.82~1.21) |  |  |  |  |  |
| Associate Chief Nurse+ | 0.28 | 0.174 | 2.584 | 0.108 | 1.32 (0.94~1.86) |  |  |  |  |  |
| **Position** |  |  |  |  |  |  |  |  |  |  |
| Clinical Nurse |  |  |  |  | 1.00 (Reference) |  |  |  |  | 1.00 (Reference) |
| (Deputy) Head Nurse | 0.326 | 0.13 | 6.306 | 0.012 | 1.39 (1.07~1.79) | 0.343 | 0.149 | 5.274 | **0.022** | **1.41 (1.05~1.89)** |
| **Education** |  |  |  | <.001 |  |  |  |  |  |  |
| Associate Degree |  |  |  |  | 1.00 (Reference) |  |  |  |  | 1.00 (Reference) |
| Bachelor's Degree | -0.185 | 0.104 | 3.183 | 0.074 | 0.83 (0.68~1.02) | -0.109 | 0.114 | 0.906 | 0.341 | 0.9 (0.72~1.12) |
| Post-graduate Degree | 0.491 | 0.216 | 5.179 | 0.023 | 1.63 (1.07~2.49) | 0.312 | 0.235 | 1.767 | 0.184 | 1.37 (0.86~2.16) |
| **Pediatric working experience（yrs）** | | | | 0.051 |  |  |  |  |  |  |
| 1~5 |  |  |  |  | 1.00 (Reference) |  |  |  |  |  |
| 6~10 | -0.113 | 0.087 | 1.716 | 0.19 | 0.89 (0.75~1.06) |  |  |  |  |  |
| 11~20 | -0.225 | 0.083 | 7.297 | 0.007 | 0.8 (0.68~0.94) |  |  |  |  |  |
| >20 | -0.044 | 0.133 | 0.112 | 0.737 | 0.96 (0.74~1.24) |  |  |  |  |  |

CH = Children’s hospitals; MCHH = Maternal and child health hospitals; GHP = General hospitals with pediatric departments; MW, Medical ward; MSW, Medical and surgical ward; SW, Surgical ward; PICU, Pediatric intensive care unit.

**Table S1d. Univariable and multivariable logistic regression analyses of factors associated with correct responses for NGT/OGT tube-tip verification timing**

| Variable | Univariable analysis | | | | | Multivariable analysis | | | | |
| --- | --- | --- | --- | --- | --- | --- | --- | --- | --- | --- |
|  | β | SE | wald χ² | P | OR (95% CI) | β | SE | wald χ² | P / omnibus | aOR (95% CI) |
| **Region** |  |  |  | 0.009 |  |  |  |  | **<.001** |  |
| Northeast |  |  |  |  | 1.00 (Reference) |  |  |  |  | 1.00 (Reference) |
| North | 0.092 | 0.082 | 1.268 | 0.26 | 1.1 (0.93~1.29) | 0.078 | 0.082 | 0.909 | 0.34 | 1.08 (0.92~1.27) |
| East | 0.232 | 0.078 | 8.889 | 0.003 | 1.26 (1.08~1.47) | 0.213 | 0.078 | 7.412 | **0.006** | **1.24 (1.06~1.44)** |
| South | 0.089 | 0.09 | 0.98 | 0.322 | 1.09 (0.92~1.31) | 0.057 | 0.091 | 0.399 | 0.528 | 1.06 (0.89~1.27) |
| Central | 0.072 | 0.083 | 0.767 | 0.381 | 1.08 (0.91~1.26) | 0.052 | 0.083 | 0.398 | 0.528 | 1.05 (0.9~1.24) |
| Northwest | 0.042 | 0.092 | 0.207 | 0.649 | 1.04 (0.87~1.25) | 0.046 | 0.092 | 0.248 | 0.619 | 1.05 (0.87~1.26) |
| Southwest | 0.039 | 0.092 | 0.183 | 0.669 | 1.04 (0.87~1.25) | 0.017 | 0.093 | 0.033 | 0.856 | 1.02 (0.85~1.22) |
| **Hospital type** |  |  |  | 0.246 |  |  |  |  |  |  |
| CH |  |  |  |  | 1.00 (Reference) |  |  |  |  |  |
| MCHH | -0.031 | 0.084 | 0.134 | 0.714 | 0.97 (0.82~1.14) |  |  |  |  |  |
| GHP | 0.094 | 0.059 | 2.547 | 0.111 | 1.1 (0.98~1.23) |  |  |  |  |  |
| **Ward** |  |  |  | <.001 |  |  |  |  |  |  |
| MW |  |  |  |  | 1.00 (Reference) |  |  |  |  | 1.00 (Reference) |
| MSW | -0.136 | 0.145 | 0.888 | 0.346 | 0.87 (0.66~1.16) | -0.158 | 0.145 | 1.193 | 0.275 | 0.85 (0.64~1.13) |
| SW | 0.148 | 0.05 | 8.89 | 0.003 | 1.16 (1.05~1.28) | 0.14 | 0.05 | 7.911 | 0.005 | 1.15 (1.04~1.27) |
| Neonatal unit | 0.255 | 0.053 | 23.441 | <.001 | 1.29 (1.16~1.43) | 0.251 | 0.053 | 22.528 | **<.001** | **1.29 (1.16~1.43)** |
| PICU | 0.259 | 0.06 | 18.381 | <.001 | 1.3 (1.15~1.46) | 0.252 | 0.061 | 17.285 | **<.001** | **1.29 (1.14~1.45)** |
| **Sex** |  |  |  |  |  |  |  |  |  |  |
| Male |  |  |  |  | 1.00 (Reference) |  |  |  |  |  |
| Female | -0.003 | 0.108 | 0.001 | 0.981 | 1 (0.81~1.23) |  |  |  |  |  |
| **Age（yrs）** |  |  |  | 0.252 |  |  |  |  |  |  |
| 18-25 |  |  |  |  | 1.00 (Reference) |  |  |  |  |  |
| 26-30 | 0.051 | 0.067 | 0.58 | 0.446 | 1.05 (0.92~1.2) |  |  |  |  |  |
| 31-35 | -0.06 | 0.066 | 0.847 | 0.358 | 0.94 (0.83~1.07) |  |  |  |  |  |
| 36-40 | -0.012 | 0.069 | 0.029 | 0.864 | 0.99 (0.86~1.13) |  |  |  |  |  |
| >40 | -0.058 | 0.08 | 0.522 | 0.47 | 0.94 (0.81~1.1) |  |  |  |  |  |
| **Professional title** |  |  |  | 0.276 |  |  |  |  |  |  |
| None |  |  |  |  | 1.00 (Reference) |  |  |  |  |  |
| Senior Nurse | 0.079 | 0.058 | 1.869 | 0.172 | 1.08 (0.97~1.21) |  |  |  |  |  |
| Supervisor Nurse | 0.102 | 0.058 | 3.126 | 0.077 | 1.11 (0.99~1.24) |  |  |  |  |  |
| Associate Chief Nurse+ | 0.163 | 0.111 | 2.145 | 0.143 | 1.18 (0.95~1.47) |  |  |  |  |  |
| **Position** |  |  |  |  |  |  |  |  |  |  |
| Clinical Nurse |  |  |  |  | 1.00 (Reference) |  |  |  |  |  |
| (Deputy) Head Nurse | 0.08 | 0.085 | 0.894 | 0.344 | 1.08 (0.92~1.28) |  |  |  |  |  |
| **Education** |  |  |  | 0.257 |  |  |  |  |  |  |
| Associate Degree |  |  |  |  | 1.00 (Reference) |  |  |  |  |  |
| Bachelor's Degree | 0.104 | 0.065 | 2.553 | 0.11 | 1.11 (0.98~1.26) |  |  |  |  |  |
| Post-graduate Degree | 0.153 | 0.156 | 0.957 | 0.328 | 1.17 (0.86~1.58) |  |  |  |  |  |
| **Pediatric working experience（yrs）** | | | | 0.565 |  |  |  |  |  |  |
| 1~5 |  |  |  |  | 1.00 (Reference) |  |  |  |  |  |
| 6~10 | 0.01 | 0.052 | 0.036 | 0.85 | 1.01 (0.91~1.12) |  |  |  |  |  |
| 11~20 | -0.029 | 0.05 | 0.332 | 0.564 | 0.97 (0.88~1.07) |  |  |  |  |  |
| >20 | -0.093 | 0.082 | 1.307 | 0.253 | 0.91 (0.78~1.07) |  |  |  |  |  |

CH = Children’s hospitals; MCHH = Maternal and child health hospitals; GHP = General hospitals with pediatric departments; MW, Medical ward; MSW, Medical and surgical ward; SW, Surgical ward; PICU, Pediatric intensive care unit.

**Table S1e. Univariable and multivariable logistic regression analyses of factors associated with correct responses for pre-insertion tube length measurement for NJT**

| Variable | Univariable analysis | | | | | Multivariable analysis | | | | |
| --- | --- | --- | --- | --- | --- | --- | --- | --- | --- | --- |
|  | β | SE | wald χ² | P | OR (95% CI) | β | SE | wald χ² | P / omnibus | aOR (95% CI) |
| **Region** |  |  |  | <.001 |  |  |  |  | **<.001** |  |
| Northeast |  |  |  |  | 1.00 (Reference) |  |  |  |  | 1.00 (Reference) |
| North | -0.114 | 0.09 | 1.59 | 0.207 | 0.89 (0.75~1.07) | -0.075 | 0.092 | 0.657 | 0.418 | 0.93 (0.78~1.11) |
| East | 0.284 | 0.084 | 11.504 | <.001 | 1.33 (1.13~1.57) | 0.329 | 0.087 | 14.334 | **<.001** | **1.39 (1.17~1.65)** |
| South | 0.583 | 0.094 | 38.222 | <.001 | 1.79 (1.49~2.16) | 0.626 | 0.098 | 41.073 | **<.001** | **1.87 (1.54~2.26)** |
| Central | -0.172 | 0.091 | 3.564 | 0.059 | 0.84 (0.7~1.01) | -0.187 | 0.092 | 4.083 | **0.043** | **0.83 (0.69~0.99)** |
| Northwest | -0.233 | 0.103 | 5.092 | 0.024 | 0.79 (0.65~0.97) | -0.184 | 0.105 | 3.078 | 0.079 | 0.83 (0.68~1.02) |
| Southwest | -0.316 | 0.104 | 9.151 | 0.002 | 0.73 (0.59~0.9) | -0.224 | 0.108 | 4.329 | 0.037 | 0.8 (0.65~0.99) |
| **Hospital type** |  |  |  | <.001 |  |  |  |  |  |  |
| CH |  |  |  |  | 1.00 (Reference) |  |  |  |  | 1.00 (Reference) |
| MCHH | -0.327 | 0.099 | 10.956 | <.001 | 0.72 (0.59~0.88) | -0.265 | 0.104 | 6.472 | **0.011** | **0.77 (0.63~0.94)** |
| GHP | 0.213 | 0.062 | 11.605 | <.001 | 1.24 (1.09~1.4) | 0.351 | 0.067 | 27.738 | **<.001** | **1.42 (1.25~1.62)** |
| **Ward** |  |  |  | <.001 |  |  |  |  |  |  |
| MW |  |  |  |  | 1.00 (Reference) |  |  |  |  | 1.00 (Reference) |
| MSW | 0.058 | 0.153 | 0.143 | 0.706 | 1.06 (0.79~1.43) | 0.027 | 0.155 | 0.03 | 0.863 | 1.03 (0.76~1.39) |
| SW | 0.113 | 0.054 | 4.37 | 0.037 | 1.12 (1.01~1.25) | 0.1 | 0.055 | 3.306 | 0.069 | 1.11 (0.99~1.23) |
| Neonatal unit | 0.01 | 0.059 | 0.028 | 0.867 | 1.01 (0.9~1.13) | -0.039 | 0.06 | 0.42 | 0.517 | 0.96 (0.85~1.08) |
| PICU | 0.566 | 0.063 | 81.826 | <.001 | 1.76 (1.56~1.99) | 0.517 | 0.064 | 66.007 | **<.001** | **1.68 (1.48~1.9)** |
| **Sex** |  |  |  |  |  |  |  |  |  |  |
| Male |  |  |  |  | 1.00 (Reference) |  |  |  |  |  |
| Female | -0.08 | 0.119 | 0.455 | 0.5 | 0.92 (0.73~1.17) |  |  |  |  |  |
| **Age（yrs）** |  |  |  | <.001 |  |  |  |  |  |  |
| 18-25 |  |  |  |  | 1.00 (Reference) |  |  |  |  | 1.00 (Reference) |
| 26-30 | -0.209 | 0.071 | 8.708 | 0.003 | 0.81 (0.71~0.93) | -0.101 | 0.086 | 1.364 | 0.243 | 0.9 (0.76~1.07) |
| 31-35 | -0.315 | 0.069 | 20.567 | <.001 | 0.73 (0.64~0.84) | -0.107 | 0.11 | 0.953 | 0.329 | 0.9 (0.72~1.11) |
| 36-40 | -0.261 | 0.073 | 12.636 | <.001 | 0.77 (0.67~0.89) | 0.037 | 0.125 | 0.085 | 0.77 | 1.04 (0.81~1.33) |
| >40 | -0.279 | 0.085 | 10.71 | 0.001 | 0.76 (0.64~0.89) | 0.087 | 0.154 | 0.317 | 0.573 | 1.09 (0.81~1.47) |
| **Professional title** |  |  |  | 0.106 |  |  |  |  |  |  |
| None |  |  |  |  | 1.00 (Reference) |  |  |  |  |  |
| Senior Nurse | -0.121 | 0.061 | 3.873 | 0.049 | 0.89 (0.79~1) |  |  |  |  |  |
| Supervisor Nurse | -0.091 | 0.062 | 2.195 | 0.138 | 0.91 (0.81~1.03) |  |  |  |  |  |
| Associate Chief Nurse+ | 0.073 | 0.118 | 0.385 | 0.535 | 1.08 (0.85~1.36) |  |  |  |  |  |
| **Position** |  |  |  |  |  |  |  |  |  |  |
| Clinical Nurse |  |  |  |  | 1.00 (Reference) |  |  |  |  |  |
| (Deputy) Head Nurse | 0.106 | 0.091 | 1.337 | 0.248 | 1.11 (0.93~1.33) |  |  |  |  |  |
| **Education** |  |  |  | 0.002 |  |  |  |  |  |  |
| Associate Degree |  |  |  |  | 1.00 (Reference) |  |  |  |  | 1.00 (Reference) |
| Bachelor's Degree | -0.137 | 0.068 | 4 | 0.046 | 0.87 (0.76~1) | -0.061 | 0.075 | 0.657 | 0.417 | 0.94 (0.81~1.09) |
| Post-graduate Degree | 0.313 | 0.159 | 3.89 | 0.049 | 1.37 (1~1.87) | 0.289 | 0.166 | 3.05 | 0.081 | 1.34 (0.97~1.85) |
| **Pediatric working experience（yrs）** | | | | <.001 |  |  |  |  |  |  |
| 1~5 |  |  |  |  | 1.00 (Reference) |  |  |  |  | 1.00 (Reference) |
| 6~10 | -0.157 | 0.056 | 7.835 | 0.005 | 0.86 (0.77~0.95) | -0.045 | 0.079 | 0.325 | 0.569 | 0.96 (0.82~1.12) |
| 11~20 | -0.217 | 0.053 | 16.565 | <.001 | 0.81 (0.73~0.89) | -0.161 | 0.101 | 2.565 | 0.109 | 0.85 (0.7~1.04) |
| >20 | -0.25 | 0.089 | 7.897 | 0.005 | 0.78 (0.65~0.93) | -0.288 | 0.16 | 3.236 | 0.072 | 0.75 (0.55~1.03) |

CH = Children’s hospitals; MCHH = Maternal and child health hospitals; GHP = General hospitals with pediatric departments; MW, Medical ward; MSW, Medical and surgical ward; SW, Surgical ward; PICU, Pediatric intensive care unit.

**Table S1f. Univariable and multivariable logistic regression analyses of factors associated with correct responses for PP tube aspiration pH interpretation**

| Variable | Univariable analysis | | | | | Multivariable analysis | | | | |
| --- | --- | --- | --- | --- | --- | --- | --- | --- | --- | --- |
|  | β | SE | wald χ² | P | OR (95% CI) | β | SE | wald χ² | P / omnibus | aOR (95% CI) |
| **Region** |  |  |  | <.001 |  |  |  |  | **<.001** |  |
| Northeast |  |  |  |  | 1.00 (Reference) |  |  |  |  | 1.00 (Reference) |
| North | -0.011 | 0.093 | 0.013 | 0.909 | 0.99 (0.83~1.19) | 0.1 | 0.096 | 1.085 | 0.298 | 1.11 (0.92~1.33) |
| East | 0.483 | 0.086 | 31.557 | <.001 | 1.62 (1.37~1.92) | 0.636 | 0.09 | 49.621 | **<.001** | **1.89 (1.58~2.26)** |
| South | 0.616 | 0.097 | 40.32 | <.001 | 1.85 (1.53~2.24) | 0.744 | 0.102 | 53.27 | **<.001** | **2.1 (1.72~2.57)** |
| Central | -0.117 | 0.094 | 1.552 | 0.213 | 0.89 (0.74~1.07) | -0.095 | 0.096 | 0.98 | 0.322 | 0.91 (0.75~1.1) |
| Northwest | -0.406 | 0.11 | 13.524 | <.001 | 0.67 (0.54~0.83) | -0.279 | 0.113 | 6.128 | 0.013 | 0.76 (0.61~0.94) |
| Southwest | -0.136 | 0.106 | 1.644 | 0.2 | 0.87 (0.71~1.07) | 0.019 | 0.11 | 0.028 | 0.866 | 1.02 (0.82~1.27) |
| **Hospital type** |  |  |  | <.001 |  |  |  |  |  |  |
| CH |  |  |  |  | 1.00 (Reference) |  |  |  |  | 1.00 (Reference) |
| MCHH | -0.311 | 0.1 | 9.688 | 0.002 | 0.73 (0.6~0.89) | -0.176 | 0.106 | 2.772 | 0.096 | 0.84 (0.68~1.03) |
| GHP | 0.232 | 0.063 | 13.64 | <.001 | 1.26 (1.12~1.43) | 0.423 | 0.068 | 38.991 | **<.001** | **1.53 (1.34~1.74)** |
| **Ward** |  |  |  | <.001 |  |  |  |  |  |  |
| MW |  |  |  |  | 1.00 (Reference) |  |  |  |  | 1.00 (Reference) |
| MSW | 0.074 | 0.155 | 0.229 | 0.632 | 1.08 (0.8~1.46) | 0.036 | 0.158 | 0.051 | 0.821 | 1.04 (0.76~1.41) |
| SW | 0.131 | 0.055 | 5.7 | 0.017 | 1.14 (1.02~1.27) | 0.104 | 0.056 | 3.389 | 0.066 | 1.11 (0.99~1.24) |
| Neonatal unit | 0.067 | 0.059 | 1.256 | 0.262 | 1.07 (0.95~1.2) | 0.025 | 0.061 | 0.165 | 0.685 | 1.03 (0.91~1.16) |
| PICU | 0.543 | 0.063 | 73.502 | <.001 | 1.72 (1.52~1.95) | 0.515 | 0.065 | 62.653 | **<.001** | **1.67 (1.47~1.9)** |
| **Sex** |  |  |  |  |  |  |  |  |  |  |
| Male |  |  |  |  | 1.00 (Reference) |  |  |  |  |  |
| Female | -0.029 | 0.119 | 0.058 | 0.81 | 0.97 (0.77~1.23) |  |  |  |  |  |
| **Age（yrs）** |  |  |  | <.001 |  |  |  |  |  |  |
| 18-25 |  |  |  |  | 1.00 (Reference) |  |  |  |  | 1.00 (Reference) |
| 26-30 | -0.078 | 0.075 | 1.074 | 0.3 | 0.93 (0.8~1.07) | -0.016 | 0.1 | 0.024 | 0.876 | 0.99 (0.81~1.2) |
| 31-35 | -0.062 | 0.073 | 0.72 | 0.396 | 0.94 (0.82~1.08) | -0.004 | 0.123 | 0.001 | 0.972 | 1 (0.78~1.27) |
| 36-40 | 0.07 | 0.076 | 0.836 | 0.36 | 1.07 (0.92~1.24) | 0.14 | 0.139 | 1.02 | 0.313 | 1.15 (0.88~1.51) |
| >40 | 0.238 | 0.086 | 7.726 | 0.005 | 1.27 (1.07~1.5) | 0.286 | 0.165 | 3.027 | 0.082 | 1.33 (0.96~1.84) |
| **Professional title** |  |  |  | <.001 |  |  |  |  |  |  |
| None |  |  |  |  | 1.00 (Reference) |  |  |  |  | 1.00 (Reference) |
| Senior Nurse | -0.008 | 0.065 | 0.016 | 0.9 | 0.99 (0.87~1.13) | -0.05 | 0.085 | 0.342 | 0.558 | 0.95 (0.81~1.12) |
| Supervisor Nurse | 0.206 | 0.064 | 10.282 | 0.001 | 1.23 (1.08~1.39) | 0.162 | 0.098 | 2.772 | 0.096 | 1.18 (0.97~1.42) |
| Associate Chief Nurse+ | 0.799 | 0.113 | 49.706 | <.001 | 2.22 (1.78~2.78) | 0.482 | 0.16 | 9.047 | **0.003** | **1.62 (1.18~2.22)** |
| **Position** |  |  |  |  |  |  |  |  |  |  |
| Clinical Nurse |  |  |  |  | 1.00 (Reference) |  |  |  |  | 1.00 (Reference) |
| (Deputy) Head Nurse | 0.695 | 0.085 | 67.238 | <.001 | 2 (1.7~2.37) | 0.448 | 0.105 | 18.306 | **<.001** | **1.57 (1.28~1.92)** |
| **Education** |  |  |  | <.001 |  |  |  |  |  |  |
| Associate Degree |  |  |  |  | 1.00 (Reference) |  |  |  |  | 1.00 (Reference) |
| Bachelor's Degree | 0.252 | 0.075 | 11.25 | <.001 | 1.29 (1.11~1.49) | 0.226 | 0.085 | 7.107 | **0.008** | **1.25 (1.06~1.48)** |
| Post-graduate Degree | 0.863 | 0.159 | 29.282 | <.001 | 2.37 (1.73~3.24) | 0.417 | 0.176 | 5.621 | **0.018** | **1.52 (1.08~2.14)** |
| **Pediatric working experience（yrs）** | | | | 0.026 |  |  |  |  |  |  |
| 1~5 |  |  |  |  | 1.00 (Reference) |  |  |  |  | 1.00 (Reference) |
| 6~10 | -0.025 | 0.058 | 0.177 | 0.674 | 0.98 (0.87~1.09) | -0.044 | 0.083 | 0.276 | 0.599 | 0.96 (0.81~1.13) |
| 11~20 | 0.053 | 0.055 | 0.93 | 0.335 | 1.05 (0.95~1.17) | -0.153 | 0.105 | 2.094 | 0.148 | 0.86 (0.7~1.06) |
| >20 | 0.219 | 0.086 | 6.422 | 0.011 | 1.25 (1.05~1.47) | -0.384 | 0.164 | 5.493 | **0.019** | **0.68 (0.49~0.94)** |

CH = Children’s hospitals; MCHH = Maternal and child health hospitals; GHP = General hospitals with pediatric departments; MW, Medical ward; MSW, Medical and surgical ward; SW, Surgical ward; PICU, Pediatric intensive care unit.

**Table S1g. Univariable and multivariable logistic regression analyses of factors associated with correct responses for flushing frequency during continuous PP tube feeding**

| Variable | Univariable analysis | | | | | Multivariable analysis | | | | |
| --- | --- | --- | --- | --- | --- | --- | --- | --- | --- | --- |
|  | β | SE | wald χ² | P | OR (95% CI) | β | SE | wald χ² | P / omnibus | aOR (95% CI) |
| **Region** |  |  |  | <.001 |  |  |  |  | **<.001** |  |
| Northeast |  |  |  |  | 1.00 (Reference) |  |  |  |  | 1.00 (Reference) |
| North | 0.293 | 0.08 | 13.57 | <.001 | 1.34 (1.15~1.57) | 0.316 | 0.082 | 14.819 | **<.001** | **1.37 (1.17~1.61)** |
| East | 0.649 | 0.076 | 73.302 | <.001 | 1.91 (1.65~2.22) | 0.683 | 0.079 | 74.431 | **<.001** | **1.98 (1.7~2.31)** |
| South | 0.983 | 0.089 | 122.937 | <.001 | 2.67 (2.25~3.18) | 0.986 | 0.092 | 114.215 | **<.001** | **2.68 (2.24~3.21)** |
| Central | 0.386 | 0.08 | 23.37 | <.001 | 1.47 (1.26~1.72) | 0.342 | 0.082 | 17.283 | **<.001** | **1.41 (1.2~1.65)** |
| Northwest | 0.099 | 0.09 | 1.213 | 0.271 | 1.1 (0.93~1.32) | 0.144 | 0.092 | 2.438 | 0.118 | 1.16 (0.96~1.38) |
| Southwest | -0.06 | 0.09 | 0.444 | 0.505 | 0.94 (0.79~1.12) | -0.036 | 0.094 | 0.148 | 0.7 | 0.96 (0.8~1.16) |
| **Hospital type** |  |  |  | <.001 |  |  |  |  |  |  |
| CH |  |  |  |  | 1.00 (Reference) |  |  |  |  | 1.00 (Reference) |
| MCHH | -0.112 | 0.081 | 1.912 | 0.167 | 0.89 (0.76~1.05) | -0.038 | 0.087 | 0.197 | 0.657 | 0.96 (0.81~1.14) |
| GHP | 0.279 | 0.058 | 23.504 | <.001 | 1.32 (1.18~1.48) | 0.388 | 0.062 | 39.449 | **<.001** | **1.47 (1.31~1.66)** |
| **Ward** |  |  |  | <.001 |  |  |  |  |  |  |
| MW |  |  |  |  | 1.00 (Reference) |  |  |  |  | 1.00 (Reference) |
| MSW | 0.007 | 0.135 | 0.003 | 0.956 | 1.01 (0.77~1.31) | -0.043 | 0.137 | 0.099 | 0.753 | 0.96 (0.73~1.25) |
| SW | 0.136 | 0.048 | 8.127 | 0.004 | 1.15 (1.04~1.26) | 0.114 | 0.049 | 5.506 | **0.019** | **1.12 (1.02~1.23)** |
| Neonatal unit | 0.077 | 0.051 | 2.248 | 0.134 | 1.08 (0.98~1.19) | 0.012 | 0.053 | 0.052 | 0.819 | 1.01 (0.91~1.12) |
| PICU | 1.121 | 0.063 | 315.673 | <.001 | 3.07 (2.71~3.47) | 1.063 | 0.064 | 274.445 | **<.001** | **2.89 (2.55~3.28)** |
| **Sex** |  |  |  |  |  |  |  |  |  |  |
| Male |  |  |  |  | 1.00 (Reference) |  |  |  |  |  |
| Female | 0.067 | 0.104 | 0.422 | 0.516 | 1.07 (0.87~1.31) |  |  |  |  |  |
| **Age（yrs）** |  |  |  | <.001 |  |  |  |  |  |  |
| 18-25 |  |  |  |  | 1.00 (Reference) |  |  |  |  | 1.00 (Reference) |
| 26-30 | -0.305 | 0.065 | 21.73 | <.001 | 0.74 (0.65~0.84) | -0.187 | 0.08 | 5.437 | **0.02** | **0.83 (0.71~0.97)** |
| 31-35 | -0.435 | 0.064 | 46.561 | <.001 | 0.65 (0.57~0.73) | -0.223 | 0.101 | 4.867 | **0.027** | **0.8 (0.66~0.98)** |
| 36-40 | -0.375 | 0.067 | 31.217 | <.001 | 0.69 (0.6~0.78) | -0.096 | 0.114 | 0.703 | 0.402 | 0.91 (0.73~1.14) |
| >40 | -0.551 | 0.077 | 50.812 | <.001 | 0.58 (0.5~0.67) | -0.138 | 0.14 | 0.978 | 0.323 | 0.87 (0.66~1.15) |
| **Professional title** |  |  |  | 0.054 |  |  |  |  |  |  |
| None |  |  |  |  | 1.00 (Reference) |  |  |  |  | 1.00 (Reference) |
| Senior Nurse | -0.1 | 0.055 | 3.319 | 0.068 | 0.91 (0.81~1.01) | -0.1 | 0.055 | 3.319 | 0.068 | 0.91 (0.81~1.01) |
| Supervisor Nurse | -0.137 | 0.055 | 6.127 | 0.013 | 0.87 (0.78~0.97) | -0.137 | 0.055 | 6.127 | **0.013** | **0.87 (0.78~0.97)** |
| Associate Chief Nurse+ | 0.022 | 0.108 | 0.043 | 0.835 | 1.02 (0.83~1.26) | 0.022 | 0.108 | 0.043 | 0.835 | 1.02 (0.83~1.26) |
| **Position** |  |  |  |  |  |  |  |  |  |  |
| Clinical Nurse |  |  |  |  | 1.00 (Reference) |  |  |  |  |  |
| (Deputy) Head Nurse | 0.069 | 0.082 | 0.694 | 0.405 | 1.07 (0.91~1.26) |  |  |  |  |  |
| **Education** |  |  |  | 0.033 |  |  |  |  |  |  |
| Associate Degree |  |  |  |  | 1.00 (Reference) |  |  |  |  | 1.00 (Reference) |
| Bachelor's Degree | -0.049 | 0.062 | 0.615 | 0.433 | 0.95 (0.84~1.08) | 0.087 | 0.069 | 1.596 | 0.206 | 1.09 (0.95~1.25) |
| Post-graduate Degree | 0.31 | 0.152 | 4.14 | 0.042 | 1.36 (1.01~1.84) | 0.325 | 0.16 | 4.12 | **0.042** | **1.38 (1.01~1.9)** |
| **Pediatric working experience（yrs）** | | | | <.001 |  |  |  |  |  |  |
| 1~5 |  |  |  |  | 1.00 (Reference) |  |  |  |  | 1.00 (Reference) |
| 6~10 | -0.237 | 0.05 | 22.11 | <.001 | 0.79 (0.71~0.87) | -0.076 | 0.071 | 1.132 | 0.287 | 0.93 (0.81~1.07) |
| 11~20 | -0.277 | 0.048 | 33.579 | <.001 | 0.76 (0.69~0.83) | -0.151 | 0.091 | 2.761 | 0.097 | 0.86 (0.72~1.03) |
| >20 | -0.491 | 0.079 | 38.941 | <.001 | 0.61 (0.53~0.71) | -0.319 | 0.144 | 4.921 | **0.027** | **0.73 (0.55~0.96)** |

CH = Children’s hospitals; MCHH = Maternal and child health hospitals; GHP = General hospitals with pediatric departments; MW, Medical ward; MSW, Medical and surgical ward; SW, Surgical ward; PICU, Pediatric intensive care unit.

**Table S1h. Univariable and multivariable logistic regression analyses of factors associated with correct responses for PP tube flushing fluid selection**

| Variable | Univariable analysis | | | | | Multivariable analysis | | | | |
| --- | --- | --- | --- | --- | --- | --- | --- | --- | --- | --- |
|  | β | SE | wald χ² | P | OR (95% CI) | β | SE | wald χ² | P / omnibus | aOR (95% CI) |
| **Region** |  |  |  | <.001 |  |  |  |  | **<.001** |  |
| Northeast |  |  |  |  | 1.00 (Reference) |  |  |  |  | 1.00 (Reference) |
| North | -0.174 | 0.089 | 3.876 | 0.049 | 0.84 (0.71~1) | -0.175 | 0.089 | 3.847 | **0.05** | **0.84 (0.71~1)** |
| East | 0.196 | 0.082 | 5.684 | 0.017 | 1.22 (1.04~1.43) | 0.202 | 0.083 | 5.873 | **0.015** | **1.22 (1.04~1.44)** |
| South | -0.24 | 0.099 | 5.9 | 0.015 | 0.79 (0.65~0.96) | -0.214 | 0.101 | 4.495 | **0.034** | **0.81 (0.66~0.98)** |
| Central | -0.081 | 0.088 | 0.85 | 0.357 | 0.92 (0.78~1.1) | -0.061 | 0.089 | 0.473 | 0.492 | 0.94 (0.79~1.12) |
| Northwest | 0.109 | 0.097 | 1.269 | 0.26 | 1.12 (0.92~1.35) | 0.09 | 0.098 | 0.846 | 0.358 | 1.09 (0.9~1.33) |
| Southwest | -0.577 | 0.106 | 29.647 | <.001 | 0.56 (0.46~0.69) | -0.549 | 0.107 | 26.331 | **<.001** | **0.58 (0.47~0.71)** |
| **Hospital type** |  |  |  | 0.088 |  |  |  |  |  |  |
| CH |  |  |  |  | 1.00 (Reference) |  |  |  |  |  |
| MCHH | -0.175 | 0.094 | 3.417 | 0.065 | 0.84 (0.7~1.01) |  |  |  |  |  |
| GHP | -0.089 | 0.066 | 1.825 | 0.177 | 0.92 (0.8~1.04) |  |  |  |  |  |
| **Ward** |  |  |  | <.001 |  |  |  |  |  |  |
| MW |  |  |  |  | 1.00 (Reference) |  |  |  |  | 1.00 (Reference) |
| MSW | 0.017 | 0.148 | 0.014 | 0.906 | 1.02 (0.76~1.36) | -0.021 | 0.149 | 0.02 | 0.889 | 0.98 (0.73~1.31) |
| SW | -0.002 | 0.053 | 0.002 | 0.966 | 1 (0.9~1.11) | -0.003 | 0.053 | 0.003 | 0.953 | 1 (0.9~1.11) |
| Neonatal unit | -0.244 | 0.059 | 17.098 | <.001 | 0.78 (0.7~0.88) | -0.233 | 0.06 | 15.21 | **<.001** | **0.79 (0.71~0.89)** |
| PICU | -0.38 | 0.07 | 29.348 | <.001 | 0.68 (0.6~0.79) | -0.383 | 0.071 | 29.164 | **<.001** | **0.68 (0.59~0.78)** |
| **Sex** |  |  |  |  |  |  |  |  |  |  |
| Male |  |  |  |  | 1.00 (Reference) |  |  |  |  |  |
| Female | -0.146 | 0.121 | 1.458 | 0.227 | 0.86 (0.68~1.1) |  |  |  |  |  |
| **Age（yrs）** |  |  |  | 0.015 |  |  |  |  |  |  |
| 18-25 |  |  |  |  | 1.00 (Reference) |  |  |  |  | 1.00 (Reference) |
| 26-30 | -0.125 | 0.072 | 3.005 | 0.083 | 0.88 (0.77~1.02) | -0.025 | 0.087 | 0.084 | 0.771 | 0.98 (0.82~1.16) |
| 31-35 | -0.23 | 0.07 | 10.668 | 0.001 | 0.79 (0.69~0.91) | -0.098 | 0.093 | 1.123 | 0.289 | 0.91 (0.76~1.09) |
| 36-40 | -0.1 | 0.074 | 1.827 | 0.177 | 0.91 (0.78~1.05) | 0.048 | 0.1 | 0.236 | 0.627 | 1.05 (0.86~1.28) |
| >40 | -0.176 | 0.086 | 4.216 | 0.04 | 0.84 (0.71~0.99) | -0.054 | 0.115 | 0.224 | 0.636 | 0.95 (0.76~1.19) |
| **Professional title** |  |  |  | 0.002 |  |  |  |  |  |  |
| None |  |  |  |  | 1.00 (Reference) |  |  |  |  | 1.00 (Reference) |
| Senior Nurse | -0.12 | 0.061 | 3.882 | 0.049 | 0.89 (0.79~1) | -0.067 | 0.076 | 0.764 | 0.382 | 0.94 (0.81~1.09) |
| Supervisor Nurse | -0.229 | 0.062 | 13.879 | <.001 | 0.8 (0.71~0.9) | -0.186 | 0.087 | 4.529 | **0.033** | **0.83 (0.7~0.99)** |
| Associate Chief Nurse+ | -0.108 | 0.121 | 0.795 | 0.373 | 0.9 (0.71~1.14) | -0.113 | 0.147 | 0.591 | 0.442 | 0.89 (0.67~1.19) |
| **Position** |  |  |  |  |  |  |  |  |  |  |
| Clinical Nurse |  |  |  |  | 1.00 (Reference) |  |  |  |  |  |
| (Deputy) Head Nurse | -0.025 | 0.094 | 0.071 | 0.79 | 0.98 (0.81~1.17) |  |  |  |  |  |
| **Education** |  |  |  | 0.7 |  |  |  |  |  |  |
| Associate Degree |  |  |  |  | 1.00 (Reference) |  |  |  |  |  |
| Bachelor's Degree | -0.058 | 0.069 | 0.712 | 0.399 | 0.94 (0.82~1.08) |  |  |  |  |  |
| Post-graduate Degree | -0.049 | 0.171 | 0.083 | 0.773 | 0.95 (0.68~1.33) |  |  |  |  |  |
| **Pediatric working experience（yrs）** | | | | 0.106 |  |  |  |  |  |  |
| 1~5 |  |  |  |  | 1.00 (Reference) |  |  |  |  |  |
| 6~10 | -0.113 | 0.057 | 3.971 | 0.046 | 0.89 (0.8~1) |  |  |  |  |  |
| 11~20 | -0.109 | 0.053 | 4.164 | 0.041 | 0.9 (0.81~1) |  |  |  |  |  |
| >20 | -0.159 | 0.089 | 3.207 | 0.073 | 0.85 (0.72~1.02) |  |  |  |  |  |

CH = Children’s hospitals; MCHH = Maternal and child health hospitals; GHP = General hospitals with pediatric departments; MW, Medical ward; MSW, Medical and surgical ward; SW, Surgical ward; PICU, Pediatric intensive care unit.

**Table S1i. Univariable and multivariable logistic regression analyses of factors associated with correct responses for PP tube-tip verification timing**

| Variable | Univariable analysis | | | | | Multivariable analysis | | | | |
| --- | --- | --- | --- | --- | --- | --- | --- | --- | --- | --- |
|  | β | SE | wald χ² | P | OR (95% CI) | β | SE | wald χ² | P / omnibus | aOR (95% CI) |
| **Region** |  |  |  | <.001 |  |  |  |  | **<.001** |  |
| Northeast |  |  |  |  | 1.00 (Reference) |  |  |  |  | 1.00 (Reference) |
| North | -0.012 | 0.092 | 0.017 | 0.897 | 0.99 (0.83~1.18) | 0.017 | 0.093 | 0.032 | 0.859 | 1.02 (0.85~1.22) |
| East | 0.41 | 0.085 | 23.023 | <.001 | 1.51 (1.27~1.78) | 0.447 | 0.088 | 25.737 | **<.001** | **1.56 (1.32~1.86)** |
| South | 0.283 | 0.098 | 8.267 | 0.004 | 1.33 (1.09~1.61) | 0.312 | 0.101 | 9.512 | **0.002** | **1.37 (1.12~1.67)** |
| Central | 0.127 | 0.091 | 1.954 | 0.162 | 1.14 (0.95~1.36) | 0.11 | 0.092 | 1.41 | 0.235 | 1.12 (0.93~1.34) |
| Northwest | -0.071 | 0.104 | 0.462 | 0.497 | 0.93 (0.76~1.14) | -0.031 | 0.105 | 0.085 | 0.771 | 0.97 (0.79~1.19) |
| Southwest | 0.004 | 0.103 | 0.001 | 0.972 | 1 (0.82~1.23) | 0.082 | 0.106 | 0.599 | 0.439 | 1.09 (0.88~1.34) |
| **Hospital type** |  |  |  | <.001 |  |  |  |  |  |  |
| CH |  |  |  |  | 1.00 (Reference) |  |  |  |  | 1.00 (Reference) |
| MCHH | -0.298 | 0.098 | 9.273 | 0.002 | 0.74 (0.61~0.9) | -0.248 | 0.102 | 5.897 | 0.015 | 0.78 (0.64~0.95) |
| GHP | 0.198 | 0.062 | 10.096 | 0.001 | 1.22 (1.08~1.38) | 0.295 | 0.066 | 20 | **<.001** | **1.34 (1.18~1.53)** |
| **Ward** |  |  |  | <.001 |  |  |  |  |  |  |
| MW |  |  |  |  | 1.00 (Reference) |  |  |  |  | 1.00 (Reference) |
| MSW | 0.054 | 0.153 | 0.123 | 0.726 | 1.06 (0.78~1.43) | 0.014 | 0.154 | 0.008 | 0.927 | 1.01 (0.75~1.37) |
| SW | 0.15 | 0.054 | 7.795 | 0.005 | 1.16 (1.05~1.29) | 0.132 | 0.054 | 5.849 | **0.016** | **1.14 (1.03~1.27)** |
| Neonatal unit | 0.052 | 0.059 | 0.778 | 0.378 | 1.05 (0.94~1.18) | 0.017 | 0.06 | 0.078 | 0.779 | 1.02 (0.91~1.14) |
| PICU | 0.478 | 0.063 | 57.279 | <.001 | 1.61 (1.43~1.82) | 0.428 | 0.064 | 45.07 | **<.001** | **1.54 (1.35~1.74)** |
| **Sex** |  |  |  |  |  |  |  |  |  |  |
| Male |  |  |  |  | 1.00 (Reference) |  |  |  |  |  |
| Female | 0.064 | 0.115 | 0.303 | 0.582 | 1.07 (0.85~1.34) |  |  |  |  |  |
| **Age（yrs）** |  |  |  | 0.002 |  |  |  |  |  |  |
| 18-25 |  |  |  |  | 1.00 (Reference) |  |  |  |  | 1.00 (Reference) |
| 26-30 | -0.14 | 0.071 | 3.858 | 0.05 | 0.87 (0.76~1) | -0.049 | 0.084 | 0.339 | 0.561 | 0.95 (0.81~1.12) |
| 31-35 | -0.249 | 0.07 | 12.659 | <.001 | 0.78 (0.68~0.89) | -0.143 | 0.107 | 1.767 | 0.184 | 0.87 (0.7~1.07) |
| 36-40 | -0.133 | 0.073 | 3.286 | 0.07 | 0.88 (0.76~1.01) | -0.012 | 0.122 | 0.01 | 0.92 | 0.99 (0.78~1.25) |
| >40 | -0.281 | 0.086 | 10.613 | 0.001 | 0.76 (0.64~0.89) | -0.003 | 0.151 | 0.001 | 0.982 | 1 (0.74~1.34) |
| **Professional title** |  |  |  | 0.757 |  |  |  |  |  |  |
| None |  |  |  |  | 1.00 (Reference) |  |  |  |  |  |
| Senior Nurse | 0.001 | 0.062 | 0 | 0.984 | 1 (0.89~1.13) |  |  |  |  |  |
| Supervisor Nurse | 0.046 | 0.063 | 0.535 | 0.465 | 1.05 (0.93~1.18) |  |  |  |  |  |
| Associate Chief Nurse+ | 0.054 | 0.121 | 0.2 | 0.654 | 1.06 (0.83~1.34) |  |  |  |  |  |
| **Position** |  |  |  |  |  |  |  |  |  |  |
| Clinical Nurse |  |  |  |  | 1.00 (Reference) |  |  |  |  |  |
| (Deputy) Head Nurse | 0.048 | 0.092 | 0.268 | 0.605 | 1.05 (0.88~1.26) |  |  |  |  |  |
| **Education** |  |  |  | 0.199 |  |  |  |  |  |  |
| Associate Degree |  |  |  |  | 1.00 (Reference) |  |  |  |  |  |
| Bachelor's Degree | 0.112 | 0.071 | 2.443 | 0.118 | 1.12 (0.97~1.29) |  |  |  |  |  |
| Post-graduate Degree | 0.237 | 0.167 | 2.023 | 0.155 | 1.27 (0.91~1.76) |  |  |  |  |  |
| **Pediatric working experience（yrs）** | | | | 0.001 |  |  |  |  |  |  |
| 1~5 |  |  |  |  | 1.00 (Reference) |  |  |  |  | 1.00 (Reference) |
| 6~10 | -0.161 | 0.056 | 8.145 | 0.004 | 0.85 (0.76~0.95) | -0.073 | 0.078 | 0.884 | 0.347 | 0.93 (0.8~1.08) |
| 11~20 | -0.112 | 0.053 | 4.483 | 0.034 | 0.89 (0.81~0.99) | -0.045 | 0.1 | 0.206 | 0.65 | 0.96 (0.79~1.16) |
| >20 | -0.32 | 0.091 | 12.343 | <.001 | 0.73 (0.61~0.87) | -0.278 | 0.16 | 3.025 | 0.082 | 0.76 (0.55~1.04) |

CH = Children’s hospitals; MCHH = Maternal and child health hospitals; GHP = General hospitals with pediatric departments; MW, Medical ward; MSW, Medical and surgical ward; SW, Surgical ward; PICU, Pediatric intensive care unit.
